# Recent Progress on Wear‐Resistant Materials: Designs, Properties, and Applications

**DOI:** 10.1002/advs.202003739

**Published:** 2021-03-24

**Authors:** Wenzheng Zhai, Lichun Bai, Runhua Zhou, Xueling Fan, Guozheng Kang, Yong Liu, Kun Zhou

**Affiliations:** ^1^ State Key Laboratory of Digital Manufacturing Equipment and Technology School of Mechanical Science and Engineering Huazhong University of Science and Technology 1037 Luoyu Road Wuhan 430074 P. R. China; ^2^ Key Laboratory of Traffic Safety on Track Ministry of Education School of Traffic and Transportation Engineering Central South University 22 South Shaoshan Road Changsha 410075 P. R. China; ^3^ State Key Laboratory of Powder Metallurgy Central South University 932 Yuelushan South Road Changsha 410083 P. R. China; ^4^ State Key Laboratory for Strength and Vibration of Mechanical Structures School of Aerospace Engineering Xi'an Jiaotong University 28 Xianning West Xi'an 710049 P. R. China; ^5^ Applied Mechanics and Structure Safety Key Laboratory of Sichuan Province School of Mechanics and Engineering Southwest Jiaotong University 111 Second Ring Road Chengdu 610031 P. R. China; ^6^ School of Mechanical and Aerospace Engineering Nanyang Technological University 50 Nanyang Avenue Singapore 639798 Singapore; ^7^ Environmental Process Modelling Centre Nanyang Environment and Water Research Institute Nanyang Technological University 1 CleanTech Loop Singapore 637141 Singapore

**Keywords:** anti‐wear applications, mechanical properties, tribology, wear‐resistant materials

## Abstract

There has been tremendous interest in the development of different innovative wear‐resistant materials, which can help to reduce energy losses resulted from friction and wear by ≈40% over the next 10–15 years. This paper provides a comprehensive review of the recent progress on designs, properties, and applications of wear‐resistant materials, starting with an introduction of various advanced technologies for the fabrication of wear‐resistant materials and anti‐wear structures with their wear mechanisms. Typical strategies of surface engineering and matrix strengthening for the development of wear‐resistant materials are then analyzed, focusing on the development of coatings, surface texturing, surface hardening, architecture, and the exploration of matrix compositions, microstructures, and reinforcements. Afterward, the relationship between the wear resistance of a material and its intrinsic properties including hardness, stiffness, strength, and cyclic plasticity is discussed with underlying mechanisms, such as the lattice distortion effect, bonding strength effect, grain size effect, precipitation effect, grain boundary effect, dislocation or twinning effect. A wide range of fundamental applications, specifically in aerospace components, automobile parts, wind turbines, micro‐/nano‐electromechanical systems, atomic force microscopes, and biomedical devices are highlighted. This review is concluded with prospects on challenges and future directions in this critical field.

## Graphical abstract

This paper provides a comprehensive review of the recent progress on structural designs, properties, and applications of wear‐resistant materials. Surface engineering and matrix strengthening strategies for the development of such materials are analyzed and relationships between their wear resistance and other mechanical properties are discussed. The review is concluded with prospects on challenges and future directions in this field.

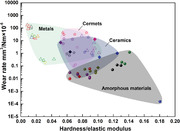

## 1 Introduction

**Figure 1:**
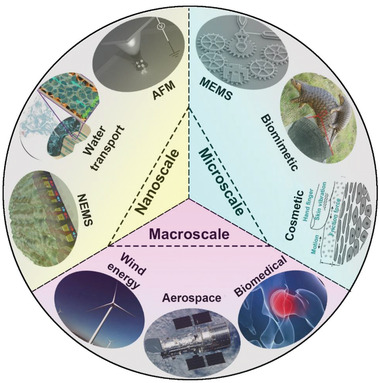
Nearly ubiquitous impact of wear in various advanced technologies from nanoscale to macroscale applications.(NEMS) Reproduced with permission.^[^ ^3a^ ^]^ Copyright 2016, American Chemical Society; (Biomimetic) Reproduced withpermission.^[^ ^3b^ ^]^ Copyright 2001, Elsevier; (Biomimetic) Reproduced with permission.^[^ ^3c^ ^]^ Copyright 2016, Elsevier; (Wind energy, Biomedical, MEMS, and Aerospace) Reproduced with permission.^[^ ^3d^ ^]^ Copyright 2015, Elsevier; (Water transport) Reproduced with permission.^[^ ^3e^ ^]^ Copyright 2017, Elsevier; (AFM) Reproduced with permission.^[^ ^3f^ ^]^ Copyright 2016, Elsevier; (Cosmetic) Reproduced with permission.^[^ ^3g^ ^]^ Copyright 2007, Elsevier.

Tribology is the study of friction, wear, and the lubrication of interacting surfaces. As one of the critical tribological domains and a persistent phenomenon regarding the deformation, damage and/or removal of material at contact surfaces, wear greatly affects the lifetime of mechanical components from the nanoscale to the macroscale.^[^ ^1^ ^]^ For example, a rapid wear of the tip of the conductive needle in an atomic force microscope (AFM) operating in a high‐current testing mode leads to low reliability in the assessment of electrical properties of the tested material. Wear debris from orthopedic joint implants is a critical cause of their failure. Its accumulation in surrounding tissues can cause detrimental tissue reactions including osteolysis and sepsis.^[^ ^2^ ^]^ The omnipresent issue of wear in a variety of advanced technologies, as shown in **Figure** **1**, continuously promotes research in high wear‐resistant materials and structures that offer effective solutions to address these challenges.

Material wear processes cover one or a combination of wear mechanisms including abrasion, fretting, adhesion, fatigue, oxidation, and other tribo‐chemical reactions.^[^ ^4^ ^]^ Wear‐resistant materials have been sought or designed to reduce material damage or loss due to wear. Typically, wear processes can be classified into three types of states, namely, low, mild, and severe wear states.^[^ ^5^ ^]^ The low wear state is normally at a stress level below the elasticity limit but can be characterized by the localized deformation of micro‐asperities and plowing.^[^ ^6^ ^]^ Micro‐cracks and localized fractures with tiny wear particles can be observed in the mild wear state,^[^ ^7^ ^]^ where the stress level is below the plasticity limit. In the severe wear state, the stress level reaches close to or above the critical failure stress.^[^ ^8^ ^]^ Crack propagation into the subsurface and macroscopic fractures with large wear flakes can be found.^[^ ^9^ ^]^

Many attempts have been made to impede the transition from low to mild wear or from mild to severe wear. As wear is a surface phenomenon, where the wear state of materials is related to their surface properties and structures, one of the most effective strategies to reduce wear is surface engineering. It contains a series of physical and/or chemical surface and subsurface modification processes but without changing the bulk properties or structures. For example, the thermochemical surface modification has demonstrated to be an effective way to strengthen the surface hardness and wear resistance of metallic materials via alloying the material surface with the C or N element by diffusion.^[^ ^10^ ^]^

Techniques used to obtain highly wear‐resistant surfaces normally involve laser processing, electro processing, mechanical processing, chemical processing, plasma processing, gas nitriding, and ion implantation.^[^ ^11^ ^]^ These techniques can be categorized into four types of approaches to increase the surface wear resistance, which include the preparation of coatings, fabrication of surface textures, development of surface hardening, and design of architecture.

In addition to surface engineering, matrix strengthening demonstrates to be an effective strategy for the enhancement of material wear resistance. In this work, matrix strengthening refers to strategies in the improvement of the wear resistance of bulk metals, ceramics, or polymers. It can be realized by composition control, microstructure design, and the addition of reinforcements. The enhancement of these approaches relies on the tailoring of deformation mechanisms^[^ ^12^ ^]^ or the introduction of hard in situ/ex situ nanoparticles.^[^ ^13^ ^]^ These particles play an important role in the improvement of the strength and hardness of the matrix. The uniformity and wettability of their distribution within the matrix affect the enhancement efficiency.

Notably, several reviews^[^ ^14^ ^]^ on hard coating focused on its preparation and tribological properties, anti‐wear polymers and their composites, self‐lubricating materials at high temperatures, etc. With the rapid development in wear‐resistant materials and their substantial impact on energy consumption, it is of great significance to offer a timely review on the recent developments of these materials. This paper presents a comprehensive review addressing critical issues related to novel designs, key properties, and wide applications of wear‐resistant materials. After the brief introduction in this section, Section 2 summarizes typical strategies in surface engineering and matrix strengthening for the development of wear‐resistant materials. In Section 3, correlations between the material wear resistance and intrinsic properties such as hardness, stiffness, strength, and cyclic plasticity are discussed together with the underlying mechanisms, including the strain hardening effect, size effect, dislocation nucleation, and grain boundary sliding. Section 4 highlights the wide applications of wear‐resistant materials in aerospace components, automobile parts, wind turbines, micro‐/nano‐electromechanical systems (MEMS/NEMS), AFM, and biomedical devices. Finally, Section 5 concludes with prospects on challenges and future directions in this critical field.

## 2 Design Strategies of Wear‐Resistant Materials

Key strategies on the improvement of material wear resistance are generally centered in surface engineering and matrix strengthening. A careful modification of the structure and composition of a surface helps to obtain high wear resistance of the surface layer compared to that of the substrate. On the other hand, good composition control and structure design within a bulk material contribute to high wear‐resistant performance without sacrificing mechanical properties of the material. Since strategies to achieve the target performance differ significantly, recent examples are presented separately in the following subsections with more details.

### 2.1 Surface Engineering

#### 2.1.1 Coatings

Wear‐resistant coatings generally enhance the surface durability in a desirable way. They have been successfully deposited on substrates by different techniques including physical vapor deposition (PVD),^[^ ^15^ ^]^ chemical vapor deposition,^[^ ^16^ ^]^ hybrid physical–chemical vapor deposition,^[^ ^17^ ^]^ and thermal spray methods.^[^ ^18^ ^]^ Comprehensive summaries on microstructures,^[^ ^19^ ^]^ characterization methods,^[^ ^14k^ ^]^ and selection criteria^[^ ^14t^ ^]^ of wear‐resistant coatings can be found in previous review works.

To achieve the high wear resistance of coatings, several strategies have been carried out including i) the ion bombardment treatment that applied prior to the coating deposition, which improves the adhesion strength due to ion‐beam‐induced interface mixing,^[^ ^20^ ^]^ ii) multiple doping of elements for strengthening the integrated coating performance, such as hardness and surface quality,^[^ ^21^ ^]^ and iii) fabrication of composite coatings with a gradient structure that efficiently enhances bonding strength at the interface between the coating and substrate.^[^ ^22^ ^]^

The hardness of coatings is strongly related to their compositions and microstructures.^[^ ^23^ ^]^ For instance, it is determined by the resistance of the coatings to bond distortion and dislocation formation and propagation, which in turn depend on the number of obstacles such as grain and column boundaries, second‐phase particles, and solutes in the coatings. Such relation has promoted coating designs, which can be achieved by tailoring microstructures. It has been clear that for nanocrystalline materials with a grain size range of 2–3 nm, the volume fraction of interfaces can approach 50%. The constitution of interfaces is important in determining the mechanical properties of coatings. The coatings with supersaturated phases embedded are for wear‐resistant applications because several pseudo‐binary nitrides or carbides show large miscibility gaps and can be fabricated by vapor deposition techniques to yield effectively‐quenched supersaturated solid solutions that are ready for age hardening.^[^ ^23^ ^]^ The coatings with optimized nanostructures and interfaces showed extremely high hardness even after thermal treatments at ≈1000 ℃.

Coating hardness could also be increased by spinodal decomposition or by the formation of precipitates that happens in several ternary, quaternary, and multinary transition metal nitrides due to their miscibility gaps.^[^ ^24^ ^]^ In high‐speed cutting applications, the so‐called self‐lubricating coatings such as diamond‐like carbon or MoS_2_ coatings are actually low‐friction protective layers formed during wear sliding.^[^ ^25^ ^]^ The better performance of these coatings can be achieved by including Mo‐containing compounds and further doping to match the lubricants used in rubbing surfaces by tuning the surface energy or to match the stiffness of the mechanical components.^[^ ^26^ ^]^ Through Ti,^[^ ^27^ ^]^ Cr,^[^ ^28^ ^]^ Zr,^[^ ^29^ ^]^ Ni,^[^ ^30^ ^]^ Ti‐Si,^[^ ^31^ ^]^ or Sb_2_O_3_^[^ ^32^ ^]^ doping, MoS_2_ coating hardness can be increased owing to a distortion of its crystal structure, which leads to improved wear resistance. Au doping of the MoS_2_ coating has a different enhancement mechanism. The good load‐supporting ability of Au nanoparticles enables MoS_2_ shearing, resulting in the superior tribological behavior of the Au‐doped MoS_2_ coating compared to that of the bare MoS_2_ coating.^[^ ^33^ ^]^

Systems with more types of elements such as ternary or quaternary systems show strong segregation in the two binary compounds with thermodynamically driven “compositional modulation,” thus resulting in isotropic coatings with enhanced mechanical and anti‐wear properties.^[^ ^34^ ^]^

The approach of self‐organization during sliding is useful in guiding the design of coatings or other wear‐resistant materials.^[^ ^35^ ^]^ Remarkable microstructural changes within the surface occur as the coatings adapt to friction and wear. Such changes are responses to the external impact, based on the principle developed by I. Prigogine,^[^ ^36^ ^]^ which states that the second thermodynamics law cannot eliminate the possibility of highly organized dissipative structures being formed in an open tribo‐system. The self‐organized structures are formed during the running‐in period of friction; their early formation can efficiently reduce the wear volume of coatings. Fox‐Rabinovich^[^ ^35^, ^37^ ^]^ pointed out that the self‐organization easily occurs for coatings with non‐equilibrium states, which could be realized by the high‐energy ion impacts in modern techniques of surface engineering such as physical vapor deposition. The non‐equilibrium states corresponding to the complexity of coatings can be tailored by methods such as element doping, the formation of solid solution and binary or ternary compounds, and the employment of multi‐layer structures. The future design of coatings should further reduce their complexity and non‐equilibrium states to make them sustain more external impacts and adapt to varying working conditions.

Recently, nanocomposite coatings have attracted intensive interest due to their exceptional physico‐mechanical properties meeting the requirements at specific working conditions, such as water lubrication and electrical contact.

Nickel‐based nanocomposite coatings have potential in applications at multiple scales due to their promising corrosion and wear resistance, good ductility, and superior electrical properties.^[^ ^38^ ^]^ For traditional metallic materials, wear resistance is related to hardness, according to the Archard theory, which depicts that wear volume *V* is inversely proportional to the hardness at a given wear condition.^[^ ^39^ ^]^ Ni‐P nanocomposite coatings were reported to show high hardness of 250–1524 HV at ambient conditions.^[^ ^40^ ^]^ It could be further improved to 1700–2000 HV by heat treatments at 200–300 °C for 30–40 weeks.^[^ ^41^ ^]^ Such improvement is due to the uniform dispersion of nickel boride formed during the low‐temperature treatments and the iron boride formed within the composite coatings.

In the past two decades, SiC, carbon nanotubes (CNTs), ferrites, nanodiamonds, and oxide nanoparticles (e.g., CeO_2_, TiO_2_, and Al_2_O_3_) have been proven to be the promising nanoadditives for the development of nanocomposite coatings.^[^ ^40^, ^42^ ^]^ Compared to SiC nanoparticles, CNTs were demonstrated to be a good additive for the enhancement of the wear resistance of Ni‐based coatings.^[^ ^43^ ^]^ They could be packed into the pores of Ni‐based coatings to form a dense passivation layer with high wear resistance. Nanocomposite coatings including CrBN, CrSiCN, CrBCN, and TiN‐/CrN‐based coatings have attracted intensive interest in water applications^[^ ^44^ ^]^ and have been reported to show good tribological properties at water lubrication conditions.^[^ ^45^ ^]^

Many studies on wear‐resistant coatings focused on the development of unique structures, for example, the core–shell structure,^[^ ^46^ ^]^ amorphous/nanocrystalline,^[^ ^47^ ^]^ and gradient multilayer coatings.^[^ ^48^ ^]^ A polymer coating with the core–shell structure of polymethylmethacrylate wrapped polytetrafluoroethylene shows a low friction coefficient of 0.069 and wear rate of 1.04 × 10^6^ mm^3^ N^−1^. Voevodin et al.^[^ ^47^ ^]^ fabricated nanocrystalline WC/amorphous DLC composite coatings with a biphasic structure composed of 1 ± 3 nm sized amorphous particles and 5 ± 10 nm sized nano‐crystal grains. The hardness of the WC/DLC coatings was much higher than that of the metal‐doped DLC coatings. The superior strength, high corrosion, and wear resistance of metallic glass make it a promising material for coatings. Sahasrabudhe et al.^[^ ^49^ ^]^ fabricated Fe‐based amorphous coatings on a Zr substrate using the laser engineered net shaping technique. The coatings consisted of crystalline phases were embedded in the amorphous matrix. The content of the amorphous phase increased after the laser heat treatment, resulting in an increase of coating hardness up to nearly 22%. After wear tests in a 3.5% NaCl solution, the wear rate of the amorphous coatings was observed to reduce by 96% compared to that of the Zr substrate. The enhancement in the wear resistance of Zr was attributed to the over 800% improvement in hardness of the Zr substrate.

The coating design with a nanocrystalline/amorphous structure is efficient in achieving excellent mechanical and tribological properties. ^[^ ^50^ ^]^ The amorphous phase can solve the problems of lattice misfits between two different polycrystalline coating materials with the random orientation of the grains. Since the lattice misfit initiates crack formation and propagation, it needs to be thin enough and form a three‐dimensional skeleton with high elastic modulus to reduce such initiation and achieve the better anti‐wear behavior of the amorphous phase. The friction coefficient of DLC coatings can reach a very low value owing to their chemical inertness and small contact area as a result of their high elastic moduli.^[^ ^51^ ^]^ The nanocrystalline phase should have a nanoscale size fitting the stability limit of the crystalline phase, which has notable tribological behavior as the nanocrystalline grains improve its hardness.

The low‐temperature deposition technique is efficient to avoid interdiffusion‐induced decreases in hardness and thus improves the coating wear resistance. The nanocrystalline/amorphous structure has been employed for the fabrication of several coatings including nc‐TiN/a‐Si_3_N_4_, nc‐TiC/a‐C, and nc‐TiCN/a‐SiCN, which improve its strength efficiently.

Existing references regarding amorphous coatings well cover research fields of fabrication processes,^[^ ^52^ ^]^ microstructures,^[^ ^53^ ^]^ mechanical behavior, corrosion,^[^ ^54^ ^]^ and wear properties.^[^ ^55^ ^]^ The wear behavior of amorphous coatings depends on the residual stress and adhesion of coatings, which are related to the difference in the thermal expansion coefficient between the coating and the substrate. The fracture toughness of coatings is a critical factor, which relates to the crack initiation and propagation during mechanical loading. Further experimental and numerical studies on adhesion, residual stress, and fracture toughness in different amorphous coating systems are required to obtain deep insights into anti‐wear mechanisms by controlling these critical factors.

Hydrogen‐free DLC coatings showed a superior anti‐wear behavior compared to hydrogenated DLC:H coatings at the water lubrication condition because delamination occurred with the latter coatings in the presence of hydrogen. ^[^ ^56^ ^]^ DLC coatings with a higher content of sp^2^‐hybridization would be promising for water lubrication. Using the a‐CN*_x_* coatings, the wear rate was maintained at an order of magnitude of 10^−7^–10^−8^ mm^3^ (Nm)^−1^ with water lubrication. Compared to the DLC and a‐CN*_x_* coatings, the TiN‐/CrN‐based coatings showed a higher wear rate of 10^–6^–10^–7^ mm^3^ (Nm)^−1^ with water lubrication but exhibited a better anti‐wear behavior under corrosive conditions owing to the formation of a passive layer during the sliding process.

Our previous studies reported the effect of sputtering ion beam energy on the bonding structures, anti‐corrosion behavior, and mechanical and tribological properties of amorphous carbon films fabricated by the ion beam sputtering method within an argon ion beam energy range of 1–3 keV. ^[^ ^57^ ^]^ Improved adhesion was obtained in the films fabricated at high ion beam energy, resulted from the collective effect of the low residual stress within the films, film graphitization, and mixed interface. The wear resistance of amorphous carbon films increased with the increase of ion beam energy. A critical parameter of hardness/elastic modulus, *H*/*E*, was demonstrated to be a suitable factor to evaluate coating wear properties.

The wear resistance and friction coefficient of coatings depend on their hardness and thickness values, respectively. The internal residual stress of coatings induced by fabrication and post‐treatment processes has an important effect on the wear resistance. The residual stress of a‐CN*_x_* coating can reach as high as 5 GPa after a PVD sputtering process, resulting in weak adhesion to the substrate. A Ti+C/a‐CN*_x_* gradient multilayer coating with a hardness of 19 GPa was prepared by Liu et al.^[^ ^48^ ^]^ using ion‐beam‐assisted magnetron sputtering. The gradient layer existed between the CN*_x_* layer and the substrate. After a thermal treatment, the Ti+C layer could alleviate the internal residual stress of the coating as a result of the formation of misfit dislocations.

Two‐dimensional (2D) materials including transition metal dichalcogenides and graphene are very attractive as the material of novel wear‐resistant coatings.^[^ ^3^, ^58^ ^]^ The typically used graphene‐based materials have high mechanical strength, good lubricity, and thermal stability, thus acting as promising coating candidates for vehicles, especially for airplanes and ships by providing light weight and high wear resistance under shear forces.^[^ ^59^ ^]^

Graphene‐based materials can mitigate mechanical failures of coatings through surface enhancement and stress transfer. The high resistance to crack initiation and deflection as well as crack branching and bridging have been determined as important strengthening mechanisms of graphene‐based materials. Graphene has been proven to be an effective solid lubricant,^[^ ^58^, ^60^ ^]^ allowing it to be a good candidate in tribological applications at both the nano‐ and the macroscale. Typically, a small amount of graphene effectively improves the wear resistance of polymer coatings.^[^ ^61^ ^]^ Meanwhile, as a monolayer material, graphene showed extraordinary anti‐wear behavior originated from interactions with hydrogen bonds to form sp^3^ carbon. ^[^ ^62^ ^]^ Nevertheless, challenges in the weak bonding strength between the metallic‐ or ceramic‐matrix and graphene‐based materials hinder practical applications of the latter materials in the fabrication of metallic and ceramic wear‐resistant coatings.

Newly emerging MXenes,^[^ ^58c^ ^]^ such as Ti_3_C_2_T*_x_*‐nanosheets, have shown ultra‐high wear resistance and good lubrication behavior under dry conditions resulted from their graphite‐like structure with low shear strength.^[^ ^63^ ^]^ A 2.3‐fold reduction in friction and a 2.7‐fold reduction in wear have been observed in the Ti_3_C_2_T*_x_*‐nanosheet modified steel surface compared with the bare steel under a contact pressure of 0.8 GPa and at relative‐low humidity. Ti_3_C_2_T*_x_*‐nanosheets show excellent anti‐friction and anti‐wear properties at moderate contact stress and low humidity conditions. Ti_3_C_2_/nanodiamonds composite MXene coatings exhibit extremely high wear resistance during sliding against a polytetrafluoroethylene counterpart owing to integrated effects of protective polytetrafluoroethylene for the 2D structure of Ti_3_C_2_, rolling ability of nanodiamonds within Ti_3_C_2_ inter/inner layers, and formation of the tribofilm.^[^ ^64^ ^]^

#### 2.1.2 Surface Texturing

Surface texturing involves the controlled modification of topography to produce functional surfaces.^[^ ^65^ ^]^ Having a good design of surface textures is also one of the most effective ways to reduce wear in MEMS/NEMS, piston‐ring systems, seals, bearings, and artificial hip implants. The surface acts as an efficient lubricant reservoir, delivering lubricants and trapping wear debris at contact surfaces.

Several mechanisms of surface texturing for the improvement of tribological behavior were reported, including i) the enhancement of load‐carrying capacity by increasing hydrodynamic pressure over the surface texture,^[^ ^66^ ^]^ ii) application of additional lubricants over the real contact area by the inlet‐suction effect,^[^ ^67^ ^]^ iii) reduction of the real contact area,^[^ ^68^ ^]^ iv) storage of lubricants via the reservoir effect,^[^ ^69^ ^]^ and v) capture of wear particles by the debris trapping effect.^[^ ^70^ ^]^

Some well‐established techniques for the fabrication of surface textures have been carried out in industries, including electro discharge machining, electrochemical processing, chemical machining, ultrasonic‐assisted machining, jet machining, and laser and beam machining. ^[^ ^71^ ^]^

Surface textures with feature sizes at micro‐ and nanoscales have been applied as secure identification elements or to improve the efficiency of solar cells but are sensitive to wear.^[^ ^72^ ^]^ Laser interference patterning demonstrates to be effective in the fabrication of the in‐volume optical grating on metallic,^[^ ^73^ ^]^ semiconductor,^[^ ^74^ ^]^ ceramic,^[^ ^75^ ^]^ and polymer^[^ ^76^ ^]^ surfaces, showing a good solution to this wear issue.^[^ ^77^ ^]^ Lasagni et al.^[^ ^78^ ^]^ fabricated line‐ and cross‐like microstructures with spatial periods of 2–7 µm in DLC layers on the steel surface by the direct laser interference patterning method. The friction coefficient reduced from 0.18 of the initial DLC layers to 0.11, which is comparable to that of the lubricated steel. By using this technique, hierarchical‐textured Ti surfaces of hole‐like structures with a spatial period of 5 µm on crater‐like structures having a spacing distance of 50 µm was obtained to decrease wear. ^[^ ^79^ ^]^ The reduced wear of the Ti surfaces was resulted from the protective effect of large craters on the small holes. The 20‐year development of direct laser interference patterning makes it a promising technique for the wear reduction of engineering applications, such as forming tools, ^[^ ^78^ ^]^ biomedical implants, ^[^ ^79^ ^]^ and decorative or security elements. ^[^ ^72^ ^]^

The precise machining of micro‐ and nano‐dimples has been considered to be an important surface texturing method to enhance the anti‐friction and anti‐wear properties of contacting surfaces in MEMS and NEMS. ^[^ ^80^ ^]^ Tang et al.^[^ ^81^ ^]^ analyzed the mechanisms of the dimples in wear reduction through a numerical model and found that a 5% dimple area fraction on the surface resulted in a wear reduction up to 72%. Sliding tests of Si_3_N_4_ mated with steel demonstrated that the tribological behavior depended on the dimple size and density rather than on the dimple shape.^[^ ^82^ ^]^ Dimples combined with a solid lubricant help in obtaining excellent tribological behavior over a wide loading range.^[^ ^83^ ^]^

A recent research study reported that the DLC‐covered textured surface improved anti‐wear properties of implant materials at high contact stresses.^[^ ^84^ ^]^ He et al.^[^ ^84b^ ^]^ found that these surfaces exhibited excellent anti‐wear behavior at the contact stress of 820 MPa. Samples with a dimple density of 24% showed a low wear rate but a high friction coefficient when compared to the surfaces with a dimple density of 44%. The improved tribological properties of these surfaces were because the hard DLC coating acted as the anti‐wear film to reduce surface wear, whereas the textured substrate prompted the lubrication behavior by providing a hydrodynamic lift effect between the sliding interfaces.

In the case of liquid lubrication, surface texturing prompts the formation of pressure‐induced tribofilms by reducing the real contact area and trapping wear debris, thus providing an additional load‐bearing capacity.^[^ ^85^ ^]^ A comprehensive review work summarizing its effects on piston rings, seals, roller bearings, and gears was carried out by Rosenkranz et al.,^[^ ^86^ ^]^ with an emphasis on rolling and sliding contact. Gropper et al.^[^ ^87^ ^]^ reported a review providing a detailed discussion on the tribological mechanisms of textured surfaces of sliding bearings from numerical and experimental points of view. **Table** **1** summarizes the information on surface textures with the corresponding tribological behavior.

**Table 1:** Microstructural parameters of dimples and test conditions and the corresponding observations for various surface textures

Material	Microstructural parameter of dimples	Test parameter	Typical observation	Ref.
Hardened steel	Diameters of 40–120 µm, depth of 5 µm, area density of 7.5–30%	Sliding speeds of 0.012–1.2 m s^−1^, contact stress of 780 MPa	Textures remained effective at severe sliding conditions	^[^ ^82^ ^]^
	Diameters of 35 and 50 µm, depths of 20–30 and 10–13 µm	Sliding speeds of 0.004–0.16 m s^−1^	Deeper and denser textures led to obvious decreases in friction and wear	^[^ ^88^ ^]^
Bearing steel	Diameters of 58 and 78 µm, depths of 5 and 5.5 µm, area density of 15% and 12%	Sliding speeds of 0.03–0.76 m s^−1^, contact stresses of 0.16–700 MPa	Regime transited from boundary to hydrodynamic lubrication at higher speeds	^[^ ^89^ ^]^
	Diameter of 60 µm and depths of 1–2 µm, grooves with pitch of 150 µm and width of 50 µm	Rolling speed of 30 m s^−1^, contact stresses of 700–2000 MPa	Friction reduced in the transverse direction	^[^ ^90^ ^]^
	Diameter of 221 µm, depth of 9.5 µm, area density of 6.7% and 26.8%	Sliding speeds of 0.01–0.12 m s^−1^, contact stress of 870 MPa	Dimples led to increases in friction at high speeds	^[^ ^91^ ^]^
	Diameter of 64 µm, depth of 16.5 µm, area density of 3.6%	Sliding speed of 0.015 m s^−1^, contact stress of 870 MPa	Textures led to a 10% increase in friction	^[^ ^92^ ^]^
	Diameter of 80 µm, depth of 5.5 µm, area density of 12%	Sliding speeds of 0.08–0.16 m s^−1^, contact stresses of 500–700 MPa	Friction reduced at high‐load and low‐sliding conditions	^[^ ^93^ ^]^
	Diameter of 200 µm, depth of 5.5 µm, area density of 7–20%	Sliding speeds of 0.09–0.55 m s^−1^, contact stresses of 260–640 MPa	Friction decreased with increasing sliding speeds and contact stresses	^[^ ^94^ ^]^
	Diameters of 20–60 µm and depths of 0.6–1.8 µm, area density of 7%	Sliding speeds of 0.09–0.55 m s^−1^, contact stresses of 390–960 MPa	Dimples with the diameter of 20 µm gave rise to decreases in friction	^[^ ^95^ ^]^
Stainless steel	Diameter of 100 µm, depth of 50 µm, area density of 40%	Sliding speed of 0.4 m s^−1^, contact stress of 1000 MPa	Friction was reduced by 50%	^[^ ^96^ ^]^

Notably, a promising strategy of integrating surface textures with coatings is attractive in the enhancement of surface tribological behavior at severe contact conditions, where both surface textures and coatings show serious wear.^[^ ^65a^ ^]^ A combination of surface texturing with soft metal,^[^ ^97^ ^]^ polytetrafluoroethylene,^[^ ^98^ ^]^ DLC,^[^ ^99^ ^]^ or 2D material^[^ ^100^ ^]^ coatings can provide low friction coefficients and wear rates. Mechanisms of each strategy are the reservoir effect of textures for metallic materials that shows the self‐lubrication behavior during sliding, formation of transfer‐films, reduction of the real contact area, and trapping effect of textures for 2D lubricant materials.

#### 2.1.3 Surface Hardening

The wear resistance of surfaces can be determined by calculating the wear volume *V* according to the Archard's law at a given condition as *V* = *KlF*/*H*, where *K* is the wear coefficient; *l* is the sliding distance; *F* represents the applied load; *H* is the hardness.

Surface hardening is a technique that commonly involves heating or using mechanical methods on the metal material to improve its surface hardness, and thus improving its resistance to wear and fatigue. Conventional methods such as carburizing, nitriding and boriding utilize element diffusion on the component surfaces need a high temperature environment. These methods mainly involve large surface‐hardened thickness and cause phase transformation in the underlayer microstructures, which increases the hardness. The latest surface hardening techniques: plasma surface diffusion,^[^ ^101^ ^]^ duplex hardening,^[^ ^102^ ^]^ laser surface alloying,^[^ ^103^ ^]^ microwave heating,^[^ ^104^ ^]^ friction stir processing,^[^ ^105^ ^]^ and high‐current pulsed electron beam processing^[^ ^106^ ^]^ are typical approaches, which are expected to induce fine microstructures or additional elements in the surface.

Studies have been devoted to surface hardening in structural applications. For example, plasma diffusion methods endowed Ti‐based alloy surfaces with improved anti‐wear properties by forming a robust surface film composed of TiC, TiN, and hard Ti‐based intermetallics.^[^ ^107^ ^]^ The duplex hardening of steel involving the conventional hardening treatment and the surface nitriding process is promising to achieve superior anti‐wear properties by increasing the surface hardness of the steel and compressive residual stresses of the subsurface.^[^ ^108^ ^]^ A slight enhancement of wear resistance was observed in M50 steel after duplex hardening because of their intrinsic carbide precipitates.

**Figure 2:**
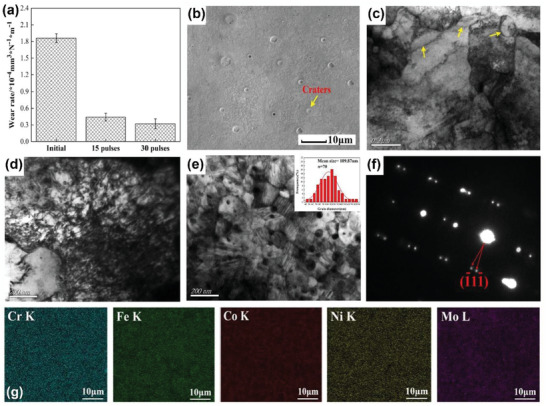
a) Wear rates of the initial and plasma diffusion modified surfaces. Plasma diffusion modified surfaces with b) craters shown by scanning electron microscopy (SEM), c) ultra‐fine grains, d) dislocations, and e,f) deformed twins shown by transmission electron microscopy (TEM) images, where the inset in (e) shows the average grain size. g) Element mapping results of plasma diffusion modified surfaces. Reproduced with permission.^[^ ^109^ ^]^ Copyright 2020, Elsevier.

As one of the efficient nonequilibrium techniques, high‐current pulsed electron beam processing has been used to modify surfaces with remarkably refined and deformed microstructures that contain the high density of crystal defects, which enhances their wear resistance and hardness. Lyu et al.^[^ ^109^ ^]^ applied the high‐current pulsed electron beam method on surfaces of CrFeCoNiMo high‐entropy alloys to realize their improved wear resistance by the underlying strengthening mechanisms. The high wear resistance and hardness were attributed to the collective effect of the high density of craters, ultra‐fine grains, dislocations, and deformed twins (see **Figure** **2a**–f). Figure 2g shows a homogeneous composition that was obtained on the modified surface after increasing the pulse number.

**Figure 3:**
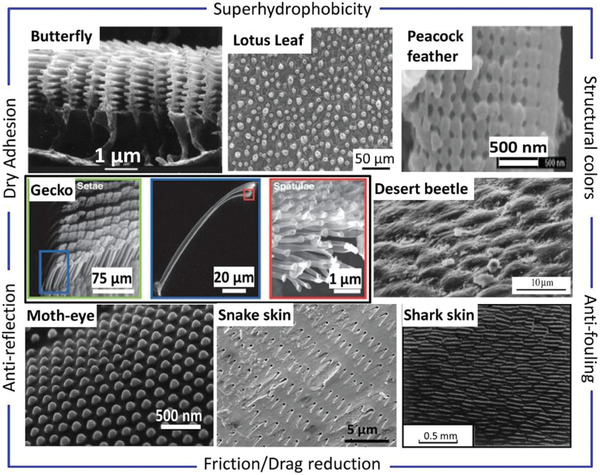
Biological hierarchical surfaces and their functionality. Reproduced with permission.^[^ ^114b^ ^]^ Copyright 2018, Elsevier.

Surface hardening has been well applied in hydrophobic surfaces to improve their durability. Garcia‐Giron et al.^[^ ^110^ ^]^ applied a low‐temperature plasma surface alloying method to improve steel surface hardness from 172 to 305 HV. Laser processing was then adopted for the fabrication of channel‐like textures with superhydrophobicity. Abrasion wear tests showed that the durability of the surfaces improved by 28–59% after the plasma carburizing process. This process enhanced mechanical properties of the surfaces, hence preventing the severe wear of the superhydrophobic surfaces.

#### 2.1.4 Architecture

Different wear mechanisms acting simultaneously over various scales and hierarchy levels result in the hierarchical and heterogeneous nature of wear. In this subsection, the design of hierarchical and heterogeneous structures on surfaces for wear reduction are discussed.

Multi‐scale textured dimples with circular and elliptical shape arrays have been fabricated on surfaces of steel to improve its tribological behavior.^[^ ^111^ ^]^ The multi‐scale textured steel surfaces showed a stabilized lower friction coefficient than the initial surface as a result of the hydrodynamic lubrication effect. The dimples with the 12% surface area density demonstrated to be the most effective in friction reduction at a normal load of 10 N and sliding speeds varying from 0.09 to 0.55 m s^−1^. The effect of multi‐scale and single‐scale textured surfaces on the tribological behavior of bearing steel has been compared by Grützmacher et al.^[^ ^112^ ^]^ Both textured samples exhibited 20–30% reduction in the friction coefficient compared to the unfeatured sample over the rotational speeds ranging from 0.033 to 0.785 m s^−1^. Specifically, the most significant reduction in friction by a factor of 4.6 was obtained in the multi‐scale textured sample where a hydrodynamic lubrication regime was formed. A mixed lubrication regime could be observed in the single‐scale textured sample. A comprehensive discussion on multi‐scale texturing in tribology can be found in a previous review by Grützmacher et al.^[^ ^113^ ^]^

Hierarchical surface engineering was initially inspired from natural phenomena and is used to adjust the hydrophobicity and adhesion on surfaces of components (**Figure** **3**).^[^ ^114^ ^]^ Lotus leaves give the famous hierarchical surface that exhibits two‐tiered roughness resulted from the superposition of two roughness patterns at different length scales.^[^ ^115^ ^]^ So far, many techniques have been developed to create multi‐scale surface roughness, such as nano‐scratching, laser surface texturing, chemical etching, and end milling, which have been well reviewed by Grützmacher et al.^[^ ^116^ ^]^ The friction reduction caused by hierarchical surfaces have been observed for materials such as titanium,^[^ ^117^ ^]^ sapphires,^[^ ^118^ ^]^ polymers,^[^ ^119^ ^]^ and composite materials.^[^ ^120^ ^]^

Several novel hierarchical structures have been fabricated in recent years to achieve remarkable adjustment in adhesion and tribology. Wang et al.^[^ ^121^ ^]^ used first‐principle calculations to analyze the nanoscale tribological behavior of fluorographene/fluorographene, MoS_2_/MoS_2_, and fluorographene/MoS_2_ heterostructures. The interlayer shear strength of the fluorographene/MoS_2_ heterostructure (2.9 MPa) was lower by almost two orders of magnitude than that of the fluorographene/fluorographene (136 MPa) and MoS_2_/MoS_2_ (470 MPa) bilayers, which was caused by the intrinsic lattice mismatch and the formation of periodic Moiré patterns, thereby leading to the extremely low energy barrier during sliding. Li et al.^[^ ^122^ ^]^ constructed the micro‐ and nanoscale binary hierarchical structure consisting of an eroded stair‐step microstructure and an inactivated nano‐particle layer on the Al surface. Wear tests demonstrated that the wear rate was reduced from 1.49 × 10^–5^ mm^2^ N^−1^ of the untreated Al surface to 0.89 × 10^–5^ mm^2^ N^−1^ of the hierarchical surface. The homogeneously‐distributed nano‐particles acting as a self‐lubricating part contributed to the friction reduction. The modified surface with hierarchical microstructures could preferentially peel off during the wear process, which helps to form a tribolayer on the surface, and thus reducing the wear.

Wu et al.^[^ ^123^ ^]^ designed hierarchical structures containing a compacted‐surface layer, a porous sub‐layer, and a barrier layer by replacing the Al cathode with graphite and by tailoring the Al^3+^ concentration. The hardness of the modified surface was 2–7 times of that of the initial Al alloy. The wear rate of the modified sample was three orders of magnitude lower than that of the untreated Al alloy, resulted from the formation of columnar wear debris that allows rolling friction on the wear surface.

Biologically‐inspired multi‐scale surface structures are attractive in friction reduction.^[^ ^124^ ^]^ Greiner et al.^[^ ^125^ ^]^ fabricated scale‐like structures with diameters varying from 13 to 150 µm on the bearing steel surface. The friction force was reduced by more than 80% compared to that of the initial steel surface at a fast sliding speed of 170 mm s^−1^. However, such structures might lead to an increase of friction of the lubricated steel‐on‐steel and steel‐on‐ceramic surfaces at a relative slow sliding speed of 20 mm s^−1^. These results stimulate further research in the field of biologically‐inspired architecture applied into anti‐friction applications.

Tuning the ratio of the elastic energy to surface energy and designing surfaces with the small‐scale roughness overlying large‐scale roughness are promising ways to reduce the friction.

### 2.2 Matrix Strengthening

Constant endeavor has been done on the preparation of bulk materials with improved wear resistance. To date, several effective strategies have centered around the control of compositions, design of microstructures, and addition of reinforcements. This subsection reviews recent experiments on matrix strengthening, focusing on its effect on anti‐wear properties.

#### 2.2.1 Compositions

Material compositions can be manipulated by additives in the matrix or by the parameter optimization of fabrication and post‐treatment processes. Dopants are widely applied in the fabrication of wear‐resistant materials. They have important effects on the lattice distortion, fracture performance, plasticity, and bonding strength of metal materials, thus influencing their wear resistance. For example, the doping of element Mo in fcc CrCoNi alloys caused the severe lattice distortion and formation of intermetallic phases, resulting in obvious improvements of compressive yield strength and hardness from 518 MPa and 244 HV to 1973 MPa and 656 HV, respectively.^[^ ^126^ ^]^ However, the plasticity of Mo‐doped CrCoNi deteriorated, caused by brittle intermetallics formed after doping. Therefore, the balance between solid solution strengthening and embrittlement is needed to achieve the suitable fracture toughness of Mo‐doped CrCoNi. By comprehensive comparisons, a suggested composition of CrCoNiMo_0.5_ was obtained, which possessed a good compromise between anti‐wear and mechanical properties.

**Figure 4:**
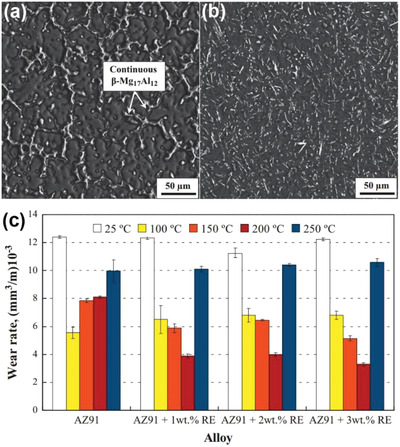
SEM images of a) initial AZ91 and b) AZ91 with 3 wt% rare earth (RE) elements. c) Wear rates of AZ91 and alloys with 1, 2, and 3 wt% RE elements after sliding 1000 m. Reproduced with permission.^[^ ^127^ ^]^ Copyright 2013, Elsevier.

Zafari et al.^[^ ^127^ ^]^ reported the effect of La‐based rare earth additions on the microstructure and anti‐wear property of AZ91 alloys at a load of 20 N and a sliding speed of 0.4 m s^−1^. The phase microstructure changed from a continuous network of the initial AZ91 alloy to the spherical morphology of the doped alloys (**Figure** **4a**,b). No obvious change in the wear resistance of the doped alloys was observed at the testing temperature of 25 ℃. As the temperature increased to 200 ℃, the wear resistance of the doped alloys was enhanced (Figure 4c). Comparing with the results of the AZ91 alloy without doping, it was implied that the decreased wear resistance originated from the *β*‐Mg_17_Al_12_ phase softening. As the test temperature increased, severe deformation of the surface and adhesion resulted. Elements Si,^[^ ^128^ ^]^ N,^[^ ^129^ ^]^ Cr,^[^ ^130^ ^]^ Co,^[^ ^129^ ^]^ Ni,^[^ ^131^ ^]^ C,^[^ ^132^ ^]^ and Ti^[^ ^133^ ^]^ have demonstrated to tune anti‐wear properties of the matrix efficiently.

**Figure 5:**
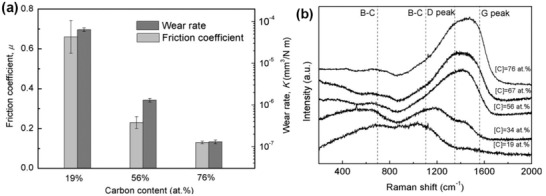
a) Friction coefficients and wear rates at a normal load of 2 N, b) Raman spectra of B_1−_ *_x_*C*_x_* films, where the intensity between 1000 and 1600 cm^−1^ wavenumber was enhanced by the increase in the amorphous carbon content. Reproduced with permission.^[^ ^134^ ^]^ Copyright 2015, Elsevier.

Despite the aforementioned doping strategy, plenty of studies have been carried out on the effects of tuning the proportion of constituent elements on the matrix wear resistance. B_1−_ *_x_*C*_x_* films of various compositions were fabricated on surfaces of Si (100) and steel substrates by controlling the electrical power of a magnetron system.^[^ ^134^ ^]^ The hardness of the B_1−_ *_x_*C*_x_* films was observed to be 28, 20, and 25 GPa, corresponding to the carbon content of 19, 56, and 76 at%. The wear rate decreased by almost two orders of magnitude with the increasing carbon content (**Figure** **5a**). This decrease was mainly caused by the presence of amorphous carbon in B_1−_ *_x_*C*_x_* films (Figure 5b), which facilitated the formation of a graphitic tribolayer. Such tailoring of the *x*‐proportion in B_1−_ *_x_*C films provides ideas in tuning anti‐wear and anti‐friction properties of bulk materials.

Similarly, Ag_1−_ *_x_*Al*_x_* alloys covering different phases were designed for potential applications in sliding electrical contact.^[^ ^135^ ^]^ An increase of the Al content enhanced the hardness of the Ag_1−_ *_x_*Al*_x_* alloys. The Al content above 25 at% led to the formation of the hexagonal close‐packed (hcp) phase, which in turn resulted in a sharp increase in the hardness of the alloys because the alloys with the hcp phase have fewer slip systems than those with the fcc phase. Thus, the former alloys showed stronger anti‐wear property than pure Ag and alloys with other phases. Since the increase of wear resistance by tuning the proportion of Al element was realized at the expense of electrical properties, further experiments and simulations are needed to develop an optimum doping composition.

#### 2.2.2 Microstructures

Similar to hierarchical or heterogeneous structure designs on surfaces discussed in Subsection 2.1.4, microstructure designs of the matrix have significant effects on anti‐wear properties of the material, which greatly depend on the grain size, precipitation (or second phases), structure of grain boundaries, dislocation, and twins in the material.

**Figure 6:**
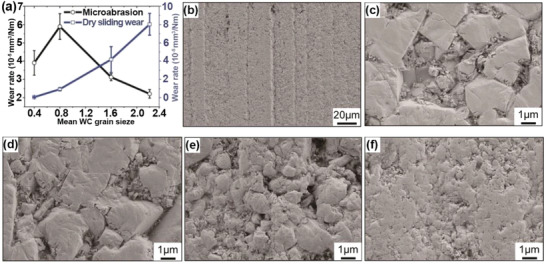
a) Wear rates of WC‐Co with various grain sizes. SEM images showing the surface morphology of WC‐Co with different grain sizes of b,c) 2.2 µm, d) 1.6 µm, e) 0.8 µm, and f) 0.4 µm after micro‐abrasion tests at a contact load of 0.2 N. Reproduced with permission.^[^ ^136^ ^]^ Copyright 2019, Elsevier.

Grain size influences the hardness of metallic materials following the classic Hall–Petch relation. Wang et al.^[^ ^136^ ^]^ reported the effect of grain sizes ranging from 0.4 to 2.2 µm on the wear resistance of WC‐Co materials. Contrary effects of grain size on the wear resistance were observed at dry sliding and micro‐abrasion conditions (**Figure** **6a**). At the dry sliding condition, the wear rate was dramatically reduced by decreasing the WC grain size, resulted from the increased hardness that mitigated the surface deformation, fracture, and oxidation. At the micro‐abrasion condition, the effect of the extraction of Co and WC grains dominated the wear mechanism over the hardness effect (Figure 6b–f). Thus, the wear resistance decreased with a decreasing grain size.

In addition to the grain size effect on the wear resistance at the microscale, the macroscale wear resistance of NiTi alloys with grain sizes ranging from 3 to 80 nm was analyzed at different normal loads by Liu et al.^[^ ^137^ ^]^ The anti‐wear performance of the nanocrystalline NiTi alloys mainly depended on the hardness. With an average grain size of 3 nm, the actual wear resistance of Ni‐W alloys was higher than the value calculated on the basis of hardness. ^[^ ^138^ ^]^ The grain growth and grain boundary relaxation led to the additional local hardening on the worn surface, thereby providing the superior wear resistance.

Suárez et al.^[^ ^139^ ^]^ prepared Ni‐based composites with the addition of 1 wt% CNTs by a hot uniaxial press method. The grain size reduced from 47.54 µm of pure Ni metals to 7.58 µm of the CNT‐reinforced composites, resulting in the improved hardness and subsequent high wear resistance of the composites. The grain refinement also enhanced the oxidation kinetics of the composites, which mitigated effects of a detrimental oxide surface layer formed, thus improving their anti‐friction behavior.

Notably, Argibay et al.^[^ ^140^ ^]^ demonstrated an inverse Hall–Petch relation of the CoCrFeMnNi high‐entropy alloy with a high wear resistance and low friction coefficient in the inert environment where oxidation was hindered. The low wear rate was observed in coarse‐grained alloys, which could be attributed to the formation of the severe deformed surface with a near‐amorphous layer. Rapidly severe plastic deformation (SPD) has been reported to occur in the coarse‐grained metallic materials during the sliding process,^[^ ^141^ ^]^ which was responsible for the change of friction and wear behavior. The low friction of pure nanocrystalline tantalum could be obtained by the formation of a nano‐grained surface layer with sizes ranging from 10 to 50 nm where grain‐boundary sliding occurred.^[^ ^142^ ^]^

The SPD treatment results in the significantly grain refinement within bulk materials. At a relative low strain level, high‐density dislocations can be induced within deformed grains, which forms cells with thick walls and low misorientation angles. As the stain level increases, the cell walls turn into high‐angle grain boundaries, and subsequently the boundaries of ultrafine grains. The hardness would thus be increased by the reduction of the grain size. However, the wear resistance of SPD‐processed bulk materials is not related to hardness alone. Their microstructures induced by different SPD processing parameters, such as the specific SPD technique, temperature, and pressure, have significant effects on the wear resistance. In this case, critical factors including the grain size, hardness, strength, ductility, strain hardening ability, oxidation rate, and strain compatibility between surface and subsurface layers should be considered for determining the wear resistance of SPD‐processed materials. A systematic discussion about the effect of these factors on wear behavior can be found in a previous study by Gao et al.^[^ ^143^ ^]^

The heterogeneous structure of different grain sizes plays a significant role in the wear resistance. Rai et al.^[^ ^144^ ^]^ designed several types of structures of steel with different grain sizes, including 4 µm, 18 µm, and a bimodal grain‐size distribution. The steel with the bimodal grain size gave the highest wear resistance as a result of the fine and strained grain effect of the heterogeneous structure. The mechanical incompatibility between various domains in other heterogeneous structures including harmonic heterogeneous lamellar structures may also significantly influence the wear resistance.

The strain hardening effect that plays a critical role in the wear resistance due to the precipitates or second phases that hinder the dislocation motion. The mechanism is discussed within the following subsection.

#### 2.2.3 Reinforcements

The application of reinforcements such as precipitates and second‐phase particles has attracted intensive attention in the fabrication of wear‐resistant bulk materials strengthened by precipitation and dispersion hardening. The wear resistance of these materials is highly dependent on their dimension, morphology, and content of reinforcements. Carbon nanomaterials including fullerenes, CNTs, graphene, and nanodiamonds show high potential as solid additives in bulk materials for high wear resistance.^[^ ^59^, ^60^, ^145^ ^]^ Emerging 2D materials such as graphene, Mxenes, hexagonal boron nitride (*h*‐BN), and transition metal dichalcogenides (TMDs) present promising enhancement capabilities owing to their high strength, large surface area, ultrathin thickness, as well as excellent thermal and chemical stability.^[^ ^58^, ^146^ ^]^

Molecular dynamics (MD) simulations revealed that van der Waals interactions between graphene and a polymer matrix could restrict surrounding polymer chains, thus providing an additional strengthening effect.^[^ ^147^ ^]^ The tribological behavior of polymers reinforced by pristine and functionalized graphene additives was compared by Li et al.^[^ ^147^ ^]^ through MD simulations. A reduction of 42.3% in wear was observed in the modified‐graphene reinforced polymers. Pull‐out processes by MD simulations demonstrated the enhanced interfacial strength between the matrix and the functionalized graphene, which was resulted from the stronger capability of the modified graphene in bonding polymer chains than that of the pristine graphene, thus leading to the less material transfer of polymers during wear.

A combination of reinforcements and ultra‐fine grained microstructures improves the anti‐wear property of metallic materials through precipitation and grain boundary hardening. Severe plastic deformation with heat treatments is a powerful method to modify metallic material structures with controllable grain size, grain boundary, dislocation density, precipitate content, and solute segregation. Recently, additive manufacturing (AM) having a high degree of freedom in designing and manufacturing demonstrates great potential in the fabrication of in situ precipitates. Wear‐resistant titanium nitrides (Ti_2_N, TiN) and N‐enriched *α* phase were observed in AM‐fabricated Ti‐based alloys.^[^ ^148^ ^]^ Microstructural characterizations of worn alloys revealed a phase transformation from *α* to *β* grains and an SPD‐induced nanocrystalline tribolayer, which resulted in an obvious reduction of wear. Precipitates nickel aluminide and chromium carbide could be formed in Ni‐18Al‐11Cr‐9C and Ni‐14Al‐8Cr‐29C alloys fabricated by laser engineered net shaping.^[^ ^149^ ^]^ As the carbon content of the alloys increased from 9 to 29 at%, the dry sliding friction coefficient remained constant. However, the wear rate of the 29 at% ones was significantly reduced as a result of the formation of more carbides that hindered the columnar grain growth.

Wang et al.^[^ ^150^ ^]^ reported an altered surface layer with a thickness of 400–800 nm on the surface of Al‐Zn‐Mg‐Cu alloys after a surface abrasion treatment. The layer consisted of ultra‐fine grains with diameters of 50–120 nm and high density of dislocations. Pre‐existing precipitates dissolved during the surface abrasion process. Precipitates of Zn, Al_2_Cu, and AlCu, which were not observed in the Al‐Zn‐Mg‐Cu alloys, formed at the top surface and grain boundaries. The enhanced diffusion motivated by vacancies, dislocations, and grain boundaries would be responsible for these newly‐formed precipitation phases.^[^ ^151^ ^]^ It can be reasonably inferred that the anti‐wear property of alloys can be changed as a result of drastic microstructural modifications of the surface. Further experiments are of importance to study effects of altering the surface layer on its tribological behavior at different working conditions.

The emerging Mxenes show the potential as reinforcement in polymers,^[^ ^152^ ^]^ metals,^[^ ^153^ ^]^ and ceramics.^[^ ^154^ ^]^ An addition of 2 wt% Mxenes in the Al‐based composite improved its bending strength and hardness by 150% and 300%, respectively, which would be beneficial in enhancing its wear resistance. Unlike the addition of other 2D materials such as MoS_2_ and graphene into metals, the addition of Mxenes into the Cu‐based composites did not facilitate the formation of a transfer layer on the contact surface.^[^ ^155^ ^]^ Further studies are needed to enhance the lubrication behavior of Mxenes as additives in bulk materials.

## 3 Properties of Wear‐Resistant Materials

Key factors affecting material wear properties include testing conditions and intrinsic material properties. Effects of testing conditions on the tribological behavior of different types of materials from the macro‐ to nano‐scale were discussed in our previous experimental and simulation studies.^[^ ^156^ ^]^ In this section, the influence of material intrinsic properties including the hardness, stiffness, strength, and cyclic plasticity on anti‐wear behavior of the materials are analyzed; correlations between the material wear resistance and these intrinsic properties are discussed.

### 3.1 Hardness

**Figure 7:**
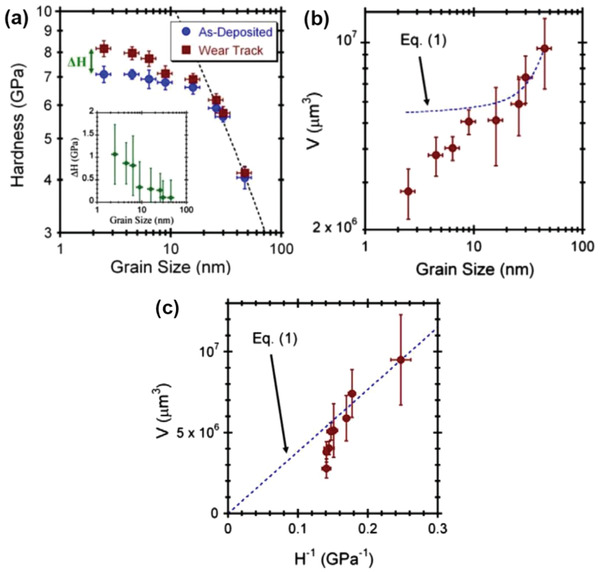
a) Hardness of specimens with different grain sizes before and after wear with the inset showing the hardness difference and dotted line representing the values obtained from the Hall–Petch relation. Wear volumes plotted against b) grain sizes and c) the corresponding hardness, where the dotted line represents values calculated on the basis of Archard theories. Reproduced with permission.^[^ ^138^ ^]^ Copyright 2010, Elsevier.

As discussed in Section 2, wear performance in materials with coarse grains correlates closely with their bulk hardness. Changing from the microcrystalline to nanocrystalline domain, as the grain size reduces, materials exhibit significantly improved wear resistance. They can be classified into two categories, depending whether their average grain size is above or below 10 nm. The wear resistance of those above 10 nm is consistent with the Archard theory.^[^ ^157^ ^]^ For those below 10 nm, an obvious deviation was observed^[^ ^138^ ^]^ and became more prominent at finer grain sizes (**Figure** **7**). Such deviations were attributed to the local hardening of the worn surface owing to the grain growth and grain boundary relaxation during the repetitive sliding.

In view of the difference between the theoretical and actual wear resistance, a linear correlation between the anti‐wear property and the ratio of wear‐related hardness *H*′over compressive elastic modulus *E* was developed by Ji et al.^[^ ^158^ ^]^ Upon the substitution of original hardness *H*, the correlation coefficient between the wear resistance and the *H*′*/E* ratio was increased from 0.61 to 0.89. The typical original hardness values, wear‐related hardness, and wear resistance results at the same test conditions are listed in **Table** **2**. Ratio *H*′^3^ */E* ^2^ is another indicative parameter of the material wear resistance for Ti‐based alloys.^[^ ^159^ ^]^

**Table 2:** Hardness, mass loss, and wear resistance of different materials,^[^ ^158^ ^]^ where the relation between wear resistance *R* and *H*′*/E* follows *R* = 2.539(1000*H*′/*E*) − 1.02_._

Material	Original hardness [HB]	Wear‐related hardness [HB]	Mass loss [mg]	Wear resistance [×10^3^ kg mm^−1^]
Al6061	53.9	59.9	5.8	0.42
Q235	127	204.5	3.4	2.06
1Cr13	162.5	260.5	2.3	3.08
0Cr19Ni9	171.3	305.7	2.6	2.75
ZGMn13	238.3	404.3	1.6	4.46
TC11	241.5	267.5	0.9	4.48
30CrMo	246.5	281.5	3.3	2.15
TC4	317	329	0.7	5.71
AlCrFeCoNiCu	335.5	441.5	2.1	3.42

The above research would be helpful in evaluating the wear performance at the wheel/rail interface, which is rarely discussed but is where severe strain hardening occurs. The strain hardening effect on the pearlitic and bainitic rail steel has been reported by Lee et al.^[^ ^160^ ^]^ The originally soft pearlitic steel was observed to be hardened more than the hard bainitic steel, thus exhibiting better wear resistance. The hardness of the pearlitic steel surface was 2.5 times higher than that of the bulk after strain hardening.^[^ ^161^ ^]^ The increase in the rail hardness simultaneously reduced the wear of the rail and wheel at nearly the same order of magnitude. At a 10% slip level, the wear of rail materials was dominated by the contact stress and was independent of the hardness.^[^ ^162^ ^]^ At higher slip levels, the frictional heat and high‐temperature hardness were the significant factors related to the wear resistance of the rail and wheel system, which calls for future efforts.

Materials with high hardness do not always show high wear resistance in practical applications. Steel tools with martensitic microstructures for high hardness cannot cut soft materials such as human hair, cheese, and potatoes, which can be attributed to the hierarchical structures of the interacting materials and the dynamic boundary conditions of their co‐deformation.^[^ ^163^ ^]^ Haircutting causes nucleation, propagation, and coalescence in the hard lath martensite. Such failure processes occur through the collective effects of i) out‐of‐plane shear stresses resulted from hair bending, ii) the asperity on the cutting edge with different microstructures on either side, and iii) contact alignments between an asperity and the outermost circumferential point of hair.

### 3.2 Stiffness

Destructive wear, characterized by a rapid increase in wear loss within a small area, may cause an increase in friction torque and a high amplitude of vibrations in mechanical components such as shafts. It thus reduces the effective structural stiffness and aggravates the stick‐slip motion.^[^ ^164^ ^]^ When the stiffness reduces to a critical range, a transition to catastrophic wear would occur at relatively low contact loading conditions. Several studies were carried out to determine the relation between the structural stiffness and wear performance.

Effects of the structural stiffness on wear behavior depend on loading forms during the impact‐sliding process. The influence of low, medium, and high structural stiffness, corresponding to 5.49, 85.83, and 686.67 × 10^–3^ N∙m^2^, respectively, on wear properties of Al‐based alloys was reported by Tan et al.^[^ ^165^ ^]^ The increase in structural stiffness resulted in a change of wear mechanisms from the delamination in the low stiffness case to the ploughing in the high stiffness case. As a result, the damaged area transferred mainly from the impact regime to the sliding regime. This was attributed to different types of dominating load determined by the structural stiffness, which would lead to changes in the stress field. It was thus concluded that the low and high structural stiffness cases exhibited counteraction effects on the wear stability, whereas the medium stiffness gave rise to the relatively stable wear.

Other than the structural stiffness, substrate stiffness has a significant effect on wear. Ding et al.^[^ ^166^ ^]^ reported effects of the stiffness of pins on the wear performance of the stainless steel/copper‐impregnated carbon in a pin‐on‐disc device. The specimens supported by elastic objects showed higher wear resistance than those supported by rigid objects with a stiffness of 2 kN m^−1^. Among stiffness values of 2–46 kN m^−1^, superior wear resistance was observed at a value of 19 kN m^−1^. At the same stiffness level, the wear resistance presented high dependence on the sliding speed. Compared to wear rates of pins supported by the rigid object, those of pins supported by elastic objects were reduced by one‐six and three‐quarters at a low speed of 8.3 m s^−1^ and a high speed of 19.4 m s^−1^, respectively.

At the nanoscale, the wear behavior of highly wear‐resistant 2D materials depends on the substrate stiffness. Different effects of the substrate stiffness have been observed from those at the macroscale. Yao et al.^[^ ^167^ ^]^ demonstrated that the substrate with high stiffness could efficiently bear normal loads and thus alleviate in‐plane stresses in the graphene supported by the substrate through restricting its deformation. The overall load‐carrying ability of the system was therefore improved. By using a well‐established finite element analysis simulation, it was reported that the load‐carrying ability of graphene‐containing systems increased monotonically with the substrate stiffness. The graphene supported by stiffer substrates exhibited higher load‐carrying ability at the nanoscale, indicating its better wear resistance. More efforts are required to analyze differences in effects of the substrate stiffness in nano‐ and macroscale domains.

### 3.3 Strength

In addition to the hardness and stiffness, strength has been reported to have an impact on the wear resistance of metallic materials. Zambrano et al.^[^ ^168^ ^]^ obtained three types of steel with the same hardness but different yield strength and strain hardening coefficients via thermal treatments, as shown in **Table** **3**. The wear performance was evaluated using a block‐on‐ring configuration at the same conditions including a sliding speed of 0.13 m s^−1^ and a contact stress of 8.14 MPa. No obvious correlation between the strain hardening coefficient and wear resistance was obtained, whereas an increase in the former raised the depth of wear‐induced deformation zones. The yield strength of the steel was related to the susceptibility of specific wear mechanisms including micro‐cutting and micro‐ploughing with high wear coefficients, which were observed in AISI O1 and AISI 1045 steel, respectively. An approximate quadratic relation was reported between the wear resistance and yield strength of the three types of steel.

**Table 3:** Summary of the yield strength, strain hardening coefficient, and roughness of the thermal‐treated steel

Steel	Yield strength [MPa]	Strain hardening coefficient	Roughness [µm]	Ref.
AISI 5160	633.96 ± 5.05	0.15 ± 0.01	0.3115 ± 0.007	^[^ ^168^ ^]^
AISI O1	386.53 ± 39.02	0.22 ± 0.01	0.2947 ± 0.005	
AISI 1045	488.80 ± 16.09	0.28 ± 0.01	0.2781 ± 0.009	
AISI 1045	530 ± 12	0.04 ± 0.01	—	^[^ ^169^ ^]^
Welded AISI 1045	415 ± 20	0.22 ± 0.02	—	
Welded AISI 1045	242 ± 11	0.39 ± 0.03	—	
Welded AISI 1045	324 ± 13	0.31 ± 0.02	—	

A similar relation between the yield strength and wear resistance was obtained by Coronado et al.^[^ ^169^ ^]^ When the hardening coefficient increases, the wear resistance decreases. Notably, the same hardness values could be achieved by tuning the yield strength and strain hardening coefficient.^[^ ^170^ ^]^ The collective effect of these two parameters on anti‐wear properties deserves more efforts.

Unlike metallic materials, the strength of polymers has diverse effects on the wear resistance. The Ratner–Lancaster plot demonstrated that the anti‐wear behavior of polymers was proportional to their tensile strength and elongation.^[^ ^171^ ^]^ By physical ageing and rejuvenation, Mergler et al.^[^ ^172^ ^]^ controlled the yield strength of polycarbonate to range from 40 to 70 MPa. Tribological and mechanical tests indicated that an increase in the yield strength alone without changing the molecular weight or density of entanglements had negligible effects on the polymer wear resistance, which depended on interactions between bonds and chains as well as the physical networks. The existing literature indicated that a single tensile property can hardly influence the wear resistance of ultra‐high molecular weight polyethylenes.^[^ ^173^ ^]^ The ratio of the maximum contact stress to yield strength *σ/σ_y_* matched well with the tendency of the wear rate and hence would be a potential coefficient to assess the polymer wear resistance.

### 3.4 Plasticity

**Figure 8:**
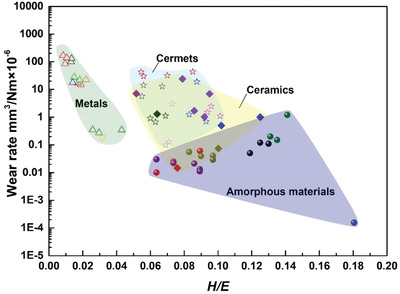
Relationship between the wear rate and *H*/*E* ratio of metals, cermets, ceramics, and amorphous materials (color‐coded).^[^ ^174^ ^]^

The ratio of hardness *H* to elastic modulus *E*, namely the “plasticity index,” is widely acceptable in determining the limit of elastic surface contact, which is critical in the wear resistance. **Figure** **8** shows the relationship between the wear rate and *H/E* ratio of metals, cermets, ceramics, and amorphous materials. In the metal, cermet, and ceramic domains, the wear rate decreases with increasing *H/E*. Typically, owing to the high ductility of metals compared to that of cermets and ceramics, a negative correlation between the wear rate and plasticity index is obtained. However, the pronounced correlation is not necessarily valid in the case of amorphous materials, where increasing wear rate is observed with increased *H/E* ratio. This exception is because a high elastic mismatch between the surface and substrate normally exists in the amorphous material case. This mismatch can induce a tensile stress at the surface/subsurface interface near the contact perimeter, which tends to strip the surface away from the substrate, thus affecting the wear resistance. It can be inferred that amorphous materials are wear‐resistant at their *H/E* range of 0.06–0.1. By adjusting the ratio of the elastic moduli of the DLC and subsurface, *E*_DLC_/*E*_Subsurface_, to a low value of 1.23, an extremely low wear rate could be obtained, indicated by the blue dot at the lower right corner of Figure 8. Thus, the control of surface elastic modulus at a value similar to that of the subsurface without an excessive decrease of the hardness is promising for the fabrication of highly wear‐resistant amorphous materials.

The *H/E* ratio has been considered to be a valuable indicator in the assessment of the abrasion wear resistance of coatings.^[^ ^174q^ ^]^ The wear resistance of Ti‐based composite coatings, such as TiN‐Al/TiN, TiN‐AlTiN/SiN, and CrTiN‐AlTiN‐AlTiCrN/SiN, increases with the increasing *H/E* ratio, ^[^ ^175^ ^]^ which is consistent with above analyses. However, TiAlN, TiAlSiN coatings and the CrN/TiAlCrSiN composite coating show no correlation between their *H/E* ratios and wear resistance. The coating thickness, preparation process, microstructure, and adhesion force between coatings and substrates are the critical factors that should be considered in further studies to evaluate the relation between the *H/E* ratio and wear resistance.

Ceramics exhibit high *H/E* ratios but are brittle because of their covalent and ionic bonds. Their wear mechanisms are determined by the contact stress. Typical stress‐related wear damages were reported by Wang et al.,^[^ ^176^ ^]^ as shown in **Table** **4**. The summary of wear mechanisms at different contact stresses provides preliminary guidelines in the selection of ceramics in anti‐wear applications.

**Table 4:** Typical stress‐related wear damages of ceramics

Contact stress	Wear damage
Low (below the elasticity limit)	Elastic deformation, no obvious wear
Medium (below the plasticity limit)	Plastic deformation‐induced wear
High (close to the critical failure stress)	Cracks‐induced wear
Very high (above the critical stress)	Fracture

**Figure 9:**
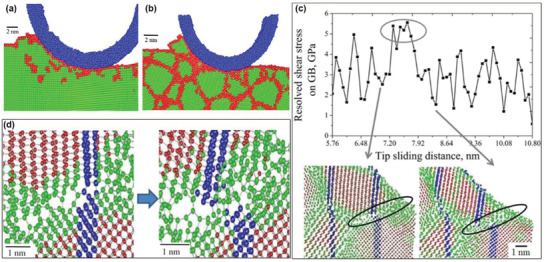
Snapshots of wear of a) single‐crystalline and b) nanocrystalline SiC sliding against a tip (blue color), where the pulling‐out of a nanocrystalline SiC grain was observed. c) Plot of the shear stress on the grain boundary against the sliding distance, where the lower panels indicate the plastic deformation in nanocrystalline SiC caused by grain boundary sliding. d) Formation of voids at grain boundaries caused by grain boundary sliding. Reproduced with permission.^[^ ^177^ ^]^ Copyright 2014, Wiley‐VCH.

Based on Table 4, anti‐wear properties of ceramics at specific operating conditions can be improved by restraining the crack initiation and propagation, which would be achieved by the reduction of grain sizes. Mishra et al.^[^ ^177^ ^]^ compared the wear resistance between nanocrystalline SiC and single‐crystalline SiC by large‐scale MD simulations that could accommodate 500 grains. The latter presented higher wear resistance because of the higher hardness originated from the dislocation‐mediated cyclic plasticity including the dislocation nucleation and motion. On the other hand, the deformation mechanism of the former was dominated by grain boundary sliding, characterized by the heterogeneous nucleation of dislocations, formation of nano‐voids, and pulling‐out of grains (**Figure** **9**). Similar effects of plastic deformation on the wear of single crystals were demonstrated in potassium bromide at the nanoscale.^[^ ^178^ ^]^ The plasticity‐related wear mechanisms could be strengthened by high normal loading and slow scratching speed conditions.

Yin and Komvopoulos^[^ ^179^ ^]^ developed a contact‐mechanical model on the basis of the slip‐line theory of plasticity to explore the wear behavior of a softer surface sliding on a harder surface. The relation between the wear resistance and material parameters including the elastic‐plastic property of soft materials, topographical feature of hard materials, and shear strength of the interface was obtained, suggesting a high dependence of the former on the *H*/*E* ratio. The relation has been demonstrated in Al_2_O_3_‐CrN, Al_2_O_3_‐TiC, and steel‐steel sliding systems, and would be promising for other ceramic‐ceramic, ceramic‐metallic, and metallic‐metallic systems.

Plastic deformation may accumulate beneath rubbing surfaces due to high cyclic stresses. Ratcheting, characterized by the directionally accumulated plastic deformation of a material, can lead to the formation of cracks on the material surface, thereby playing a significant role in detecting wear and fatigue behaviors. A cyclic plasticity material model was developed to evaluate the ratcheting behavior of the rail steel under cyclic rolling contact.^[^ ^180^ ^]^ Crack initiation occurred when the ratcheting strain reached the ductility limit of the steel. The ratcheting strain rate and the ductility limit were thus determined as the critical factors that played a deterministic role in the crack initiation lifetime. The steel with a low carbon content of 0.85% showed the consistent crack initiation lifetime at a wide loading condition and better ratcheting resistance to damage compared to that with a carbon content of 1%, thus presenting high wear and fatigue resistance. These results obtained from the model match those of the in‐service rail steel. Potential strategies can therefore be derived to alleviate the rail wear.

## 4 Applications of Wear‐Resistant Materials

The incorporation of innovative materials and advanced processing techniques can enhance the strength, hardness, toughness, and anti‐wear performance of the surface, which are critical to maintain durability, stability, and accuracy, and to enhance the wear resistance and efficiency of mechanical parts from the macroscale to nanoscale. This section focuses on the recent progress on employing innovative wear‐resistant materials in a broad range of applications, with an emphasis on aerospace components, automobile parts, wind turbines, MEMS/NEMS, AFM, and biomedical devices.

### 4.1 Aerospace Components

Materials used in aerospace components exhibit desirable mechanical properties, reasonable density for weight reduction, good damage tolerance for prolonged utilization at the demanding temperature of −30–600 ℃ and radiation conditions.^[^ ^181^ ^]^ The widely applied materials for aerospace components include Al‐, Mg‐, Ti‐, and Ni‐based alloys.^[^ ^181^, ^182^ ^]^ Wear, specifically the fretting wear incorporating synchronic and competitive processes of material failure by wear, corrosion, and fatigue, is one of the major concerns in bearing shafts, bolted connections, and blade‐disc assembly applications. An acceptable way to overcome this challenge is the use of thermostable wear‐resistant coatings with effective reinforcements over the alloy bulk.^[^ ^181a^ ^]^

**Figure 10:**
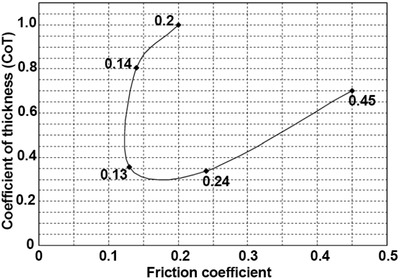
Relation between the coefficients of friction and coating thickness, where CoT denotes the ratio of the average thickness during wear to the initial thickness. Reproduced with permission.^[^ ^183^ ^]^ Copyright 2008, Elsevier.

A thermally‐sprayed double‐layer system was determined as an effective wear‐resistant coating of Ti‐based alloys.^[^ ^183^ ^]^ Prior to the coating on a TiAl6V4 alloy, a 100–120 µm thickness metallic interlayer film consisting of Cu, In, and Ni elements was deposited on the substrate by plasma spraying, which acted as a bonding layer. A 30–50 µm thickness wear‐resistant coating composed of MoS_2_ and epoxy resin was then applied on the metallic film, yielding a double‐layer coating with a total thickness of ≈150 µm. The thickness and friction coefficient played a deterministic role in the identification of fretting wear with four stages including running‐in, steady‐wear, coating‐deterioration, and terminal‐wear processes. A decreased friction coefficient accompanied with reduced coating thickness was observed at the running‐in process. During the steady‐wear process, the coating was progressively consumed at a stable friction coefficient, which was the critical factor for determining the coating durability. The friction coefficient increased at the coating‐deterioration stage with limited thickness reduction caused by the contamination of the coating. At the terminal‐wear stage, both the friction coefficient and coating thickness increased as a result of debris accumulation. A typical relation between the friction coefficient and relative coating thickness is presented in **Figure** **10**.

The reinforcement content, particle, size, and distribution uniformity have a significant effect on the wear resistance of the coatings. A homogeneous distribution of nanoparticles or hybrid reinforcement particles within the matrix show an outstanding enhancement effect.^[^ ^184^ ^]^ Ranjith et al.^[^ ^185^ ^]^ developed high‐performance Al‐Zn‐Mg‐Cu alloys by the addition of 7.5 wt% boron carbide and 2.5/5/7.5/10 wt% silicon carbide. Potassium titanium fluoride was used to enhance the bonding between the matrix and the reinforcement, which resulted in the superior wear resistance and strength of the alloy with 2.5 wt% boron carbide.

### 4.2 Automobile Parts

Steel and aluminum alloys are the workhorse materials of transportation owing to their good mechanical properties and relatively low cost.^[^ ^186^ ^]^ The transportation industry contains ≈1600 million vehicles ^[^ ^187^ ^]^ including road vehicles, trucks, trains, ships, and airplanes. Globally, ≈83 EJ of energy is used in the road vehicle sector every year, of which 32% is used to overcome friction. The percentage of energy used to overcome friction in other transportation sectors is estimated to be ≈30%.^[^ ^188^ ^]^ The energy consumption caused by wear is 10% of that used to overcome friction. The implementation of novel wear‐resistant materials and advanced lubrication technologies in transportation reduces the wear loss for vehicles because of fewer component replacements and remanufacturing issues involved. Tremendous efforts have been developed in exploring new anti‐wear materials and structures to reduce the friction and wear over the past decades, which may be referred to ref. ^[^ ^189^ ^]^.

**Figure 11:**
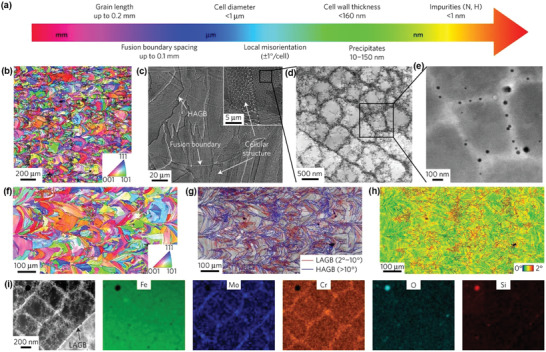
a) Diagrammatic sketch of the AM 316L stainless steel at various length scales. b) Electron‐backscatter diffraction (EBSD) inverse‐pole figure (IPF) and c) SEM images of AM specimens, showing grain orientations and unique cellular structures. d) TEM image of the unique cells. e) High‐angle annular dark‐field scanning TEM image of the cells. f) EBSD IPF image obtained at a 1 µm step size. g) EBSD image quality map showing low‐angle grain boundaries. h) Kernel average misorientation image showing the misorientation. i) TEM images of the cells with the energy dispersive spectrum mapping. Reproduced with permission.^[^ ^190^ ^]^ Copyright 2018, Springer Nature.

AM, which enables desirable flexibility in designing and manufacturing, is promising in fabricating highly wear‐resistant materials used in vehicle components. In the rest of this subsection, potential applications of high‐performance AM metallic materials in wear reduction are discussed. By AM, Wang et al.^[^ ^190^ ^]^ fabricated the austenitic 316L stainless steel with both high yield strength and tensile ductility, surpassing those of the conventional 316L steel, where an increase of the strength typically reduces the ductility. The high yield strength was attributed to unique cellular structures, low‐angle grain boundaries, and AM‐induced dislocations (**Figure** **11**). The superior ductility was related to the formation of hierarchically‐heterogeneous microstructures that caused the steady and progressive strain hardening. Effects of dislocations and progressive strain hardening are critical in the enhancement of the wear resistance of metallic materials as discussed in Subsections 2.2 and 3.1.

**Figure 12:**
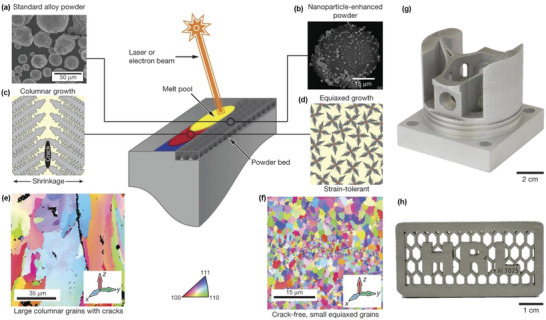
Overview of the AM process presented by the central diagrammatic sketch. a) Conventional Al powder. b) Nanoparticle‐modified Al powder. c) Typical columnar growth of dendrites during AM. d) Nanoparticle‐induced uniform nucleation. e) Typical large grains and cracks by traditional AM methods. f) Fine‐equiaxed grains in the nanoparticle‐modified powder. g) Typical AM Al piston. h) AM‐manufactured Al HRL logo. Reproduced with permission.^[^ ^191^ ^]^ Copyright 2017, Springer Nature.

The introduction of zirconium nanoparticles as crystal nuclei helped in the AM‐fabrication of crack‐free aluminum alloys with fine‐grained microstructures (**Figure** **12**), leading to their high strength comparable to that of wrought counterparts.^[^ ^191^ ^]^ The zirconium nanoparticles were reported to preferentially react with Al element in the melting pool to form the Al_3_Zr phase, which has more than 20 matching interfaces with the Al phase, thus providing low‐energy nucleation sites. A good combination of the high strength and ductility could be obtained in AM‐fabricated Ti‐based alloys that exhibited the ultrafine eutectoid microstructure, resulted from the high cooling rate and multiple thermal cycling during the AM process.^[^ ^192^ ^]^ These novel AM methods would be applied in the fabrication of complex‐shaped vehicle components, where solidification cracking is commonly observed in conventional counterparts.

### 4.3 Wind Turbines

Fretting, abrasion, and erosion wear are the important factors for evaluating the performance of wind turbines, where the blades are subjected to high loading conditions and exposed to harsh environments. Statistically, wear decreases the efficiency up to 25% in the first two‐year period.^[^ ^193^ ^]^ A number of wear‐resistant coatings such as Co‐, Ni‐, and Ni_3_Al‐based alloy coatings have been explored to protect the surface from severe wear. The high wear resistance of Co‐based alloy coatings correlates to the formation of hard phases within their microstructures such as chromium, tungsten, molybdenum, and niobium carbides, while the exceptional wear resistance of Ni‐ and Ni_3_Al‐based alloy coatings is attributed to the addition of a low Si content acting as a refiner and thus reducing the grain size. An addition of rare earth metals in the Co‐based alloy coatings decreases the brittleness.

**Figure 13:**
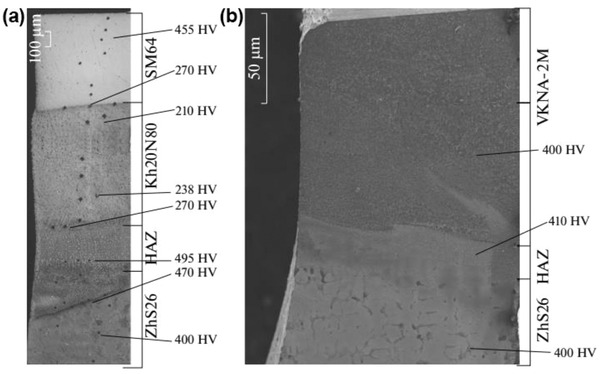
Structures with the indicated hardness of a) Ni superalloy + hard alloy composite and b) Ni_3_Al‐based alloy coatings, where ZhS26 was the substrate; HAZ denotes the heat‐affected zone; Hh20N80, SM64, and VKNA‐2M are Ni superalloy, hard alloy, and Ni_3_Al‐based alloy, respectively. Reproduced with permission.^[^ ^194^ ^]^ Copyright 2019, Springer Nature.

Abraimova et al.^[^ ^194^ ^]^ fabricated a highly resistant Ni superalloy and hard alloy composite coatings and Ni_3_Al‐based alloy coatings on the turbine blades by the welding method with a non‐consumable electrode under argon protection. The deposited crack‐free coatings showed a gradient in hardness across the coating thickness (**Figure** **13**), which would enhance the wear resistance and mechanical behavior of the turbines. Compared to the composite coatings, the single‐phase hard alloy coatings suffered from the earlier cracking caused by the insufficient ductility. Up till now, detailed data about wear‐resistant materials utilized in turbines are limited.

### 4.4 Micro‐/Nano‐Electromechanical Systems

A high surface‐area‐to‐volume ratio extends the lifetime of MEMS/NEMS devices as adhesion and friction are reduced. Silicon is commonly utilized in MEMS/NEMS but experiences high friction and wear during sliding contact owing to the formation of an oxide layer.^[^ ^195^ ^]^ Wear resistance at the nanoscale is one of deterministic factors in influencing the efficiency and reliability of MEMS/NEMS assemblies. The application of thin lubricant layers with high elastic moduli and hardness on MEMS/NEMS assembly surfaces is an accepted method in improving their wear resistance. DLC coatings, one of the mostly accepted hard films, has been successfully applied in MEMS/NEMS devices.^[^ ^196^ ^]^ Alternatively, lubricant layers with molecular‐level thickness and relative strong bonding to the MEMS/NEMS surfaces are desirable.^[^ ^197^ ^]^ Perfluoropolyether is expected to be a lubricating candidate in the magnetic disk drive of MEMS/NEMS because of its advantages of outstanding lubricity, low surface tension as well as high thermal and chemical stability.^[^ ^198^ ^]^ Thermal‐treated perfluoropolyether coatings enabling partial bonding with the substrate can provide superior wear resistance for the substrate compared to untreated coatings with complete bonding. The improved anti‐wear property resulted from the lubricant replenishment effect of unbonded coatings.

Owing to their high electrical and thermal conductivities, ionic liquids have been demonstrated to be an effective lubricant for MEMS/NEMS devices. Ionic liquid films showed a dependence of the anti‐wear property and durability on microstructures of anionic molecules, wettability, and ambient conditions.^[^ ^199^ ^]^ Four types of ionic liquid films containing different anions including hexafluorophosphate, tetrafluoroborate, nitrate, and perchlorate were prepared on the monocrystalline silicon substrate via a dip‐coating method.^[^ ^200^ ^]^ The ionic liquid film containing hexafluorophosphate presented the best wear resistance among the four, making it a possible candidate to enhance the wear resistance of MEMS/NEMS.

The combination of a thin film and micro‐patterning is another strategy to enhance the tribological performance of MEMS/NEMS. Zhao et al.^[^ ^195^ ^]^ fabricated micro‐grooves on DLC‐ionic liquid films by plasma etching, magnetron sputtering, and dip‐coating techniques. The obviously reduced friction of the films with patterns was observed. Micro‐grooves reducing the real contact area and thin ionic liquid films avoiding the direct contact between the DLC layer and the sliding tip were the causes.

### 4.5 Atomic Force Microscopes

By detecting the interaction between its tip and a specimen, AFM can characterize the topography of the specimen surface and the stress state of the interface at the nanoscale. AFM tips always suffer from wear caused by friction, typically at high‐current testing conditions. In a broad velocity range up to 100 mm s^−1^, tip wear showed a logarithmic dependence on the sliding velocity.^[^ ^201^ ^]^ For specific probes, such as the silicon nitride probe, wear can be aggravated with an increase of humidity. Ultrananocrystalline diamond probes with 30–40 nm radii, smooth surfaces, and controllable geometries were fabricated by Liu et al.^[^ ^202^ ^]^ In contrast with the conventional silicon nitride probes with similar geometries, the ultrananocrystalline diamond probes demonstrated superior wear resistance at humidity conditions of 15% and 70% relative humidity (RH) and at a wide range of stresses of 2–8 GPa. An energy model for the evaluation of probe radii identified that the ultrananocrystalline diamond probes possessed ultra‐high wear resistance which was almost ten times higher than that of the commercial silicon nitride probes. Detailed information about the energy model may be referred to ref. ^[^ ^203^ ^]^. For a detailed comparison, **Table** **5** summarizes the wear behavior of AFM probes fabricated using different materials that show different wear results with several orders of magnitude. This summary would help in the selection of AFM probes with desirable anti‐wear properties at specific testing conditions.

**Table 5:** Summary of materials, wear data, and testing conditions for different types of AFM probes

Material (probe‐specimen)	Testing condition	Wear behavior	Ref.
SiN*_x_*‐Si	10 nN, 10 m s^−1^, 25.6 mm	Fatigue wear	^[^ ^204^ ^]^
	100 nN, 20 m s^−1^, 72 mm	Adhesive, abrasive, and fatigue wear with a wear rate of 5 × 10^–2^ mm^3^ (Nm)^−1^	^[^ ^205^ ^]^
Si‐SiO_2_	1–6 µN, 20 µm s^−1^, KOH solution	Tribochemical wear with wear rates of 10^–4^–10^–2^ mm^3^ (Nm)^−1^, depending on the pH of the KOH solution	^[^ ^206^ ^]^
SiN*_x_*‐glasses/quartz/sapphires/ mica/SiN*_x_*/metal oxides	1–6 N, 16 m s^−1^, 67.5 mm sliding distance, water, NH_4_OH, and HCl solutions	Tribochemical wear independent of pH	^[^ ^207^ ^]^
Si/SiN*_x_*‐Si	10–300 nN, 5 m s^−1^, 2.56–25.6 mm, 20–60% RH	Wear rates of 10^–4^–10^–2^ and 10^–6^–10^–5^ mm^3^ (Nm)^−1^ for Si and SiN*_x_* probes, respectively, which increased with the increasing RH	^[^ ^208^ ^]^
Si‐Si/DLC	10–100 nN, 1 m s^−1^, 0.032–70 mm, air and N_2_	Wear rates of 10^–5^–10^–3^ mm^3^ (Nm)^−1^ depending on the environmental conditions	^[^ ^209^ ^]^
Si‐piezoelectric ceramic materials	10–50 N, 2 m s^−1^, 1.6–18 mm	Wear rates of 10^–5^–10^–3^ mm^3^ (Nm)^−1^ depending on the probe shapes	^[^ ^210^ ^]^
Si‐polymers	5–100 nN, 1.5 mm s^−1^, 750 m, vacuum	Exponential wear rate dependent on the contact stresses	^[^ ^211^ ^]^
Si/SiN*_x_* coated Si‐ultrananocrystalline diamonds	0 N, 3.97 m s^−1^, 102.4 mm, ambient conditions	Failure of Si probes and SiN*_x_* coatings	^[^ ^212^ ^]^
SiC/Si‐polymers/ SiO_2_	2.5–10 nN, 1.5 mm s^−1^, 40–100 m, vacuum and ambient conditions	Higher wear resistance of SiC probes	^[^ ^213^ ^]^
Si‐diamonds	0 N, 4, 21 nm s^−1^, 0.2–4.5 m, vacuum conditions	Wear volume of 25 nm^3^	^[^ ^214^ ^]^
Carbon‐coated probes‐Si/SiO_2_	—	Higher wear resistance of carbon coated probes	^[^ ^215^ ^]^
Diamonds‐Si	60 N, 2 m s^−1^, 7.68 mm	Wear rate of 1.9 × 10^–9^ mm^3^ (Nm)^−1^	^[^ ^216^ ^]^
CNT‐polycrystalline Si	2 m	Higher wear resistance of CNT probes (20 times more than that of Si probes)	^[^ ^217^ ^]^
Polycrystalline diamond coated probes‐Cu/Si/SiN*_x_*	1–5 N, 1 m s^−1^, 0.3–1.5 mm	Wear rate of 10^–7^–10^–6^ mm^3^ (Nm)^−1^	^[^ ^218^ ^]^
Ultrananocrystalline diamonds‐ultrananocrystalline diamonds	0–100 nN, 3.75–20.3 m s^−1^, 1.024–204.8 mm, 15% and 70% RH	Ultrahigh wear resistance of ultrananocrystalline diamond probes	^[^ ^202a^ ^]^
Si‐doped DLC‐SiO_2_	1–17.5 nN, 10–250 m s^−1^, 2 m, ambient conditions	Wear rates of 10^–7^–10^–5^ mm^3^ (Nm)^−1^	^[^ ^219^ ^]^
Ti/Pt/Au coated probes‐SiO_2_ and highly oriented pyrolytic graphite	Electric contact at ambient conditions	Rapid wear	^[^ ^220^ ^]^
Au/Pt/Ir coated probes‐Cu and highly oriented pyrolytic graphite	Electric contact at ultra‐high vacuum conditions	Weakness of metal‐coated probes to the lateral force and melting of the coating layer	^[^ ^220^ ^]^
Pt‐coated probes‐DLC	50–100 nN, 0.1–100 mm s^−1^, 2 m	Adhesive, abrasive, and tribochemical wear at a logarithmic wear rate, depending on the sliding speeds	^[^ ^201^ ^]^
Pt/AuNi/PtIr/PtNi coated probes‐piezoelectric ceramic materials	1–80 nN, 10–100 mm s^−1^, 100–300 m	Wear rate of 10^–7^ mm^3^ (Nm)^−1^	^[^ ^221^ ^]^

### 4.6 Biomedical Devices

Implant materials within the body would be corroded by reduced pH during inflammation in a low oxygen and high salinity environment.^[^ ^222^ ^]^ Fretting or wear accelerates the damage of protective oxide films of the implant parts.^[^ ^223^ ^]^ Ions of V, Ni, Co, and Cr would thus be released, giving rise to cytotoxic and genotoxic effects.^[^ ^224^ ^]^ Wear debris with a particle size of ≈0.05 µm can be produced within the interface between the femoral head and the cup of hip implants, which would be identified as invasive objects, thereby resulting in an inflammatory symptom.^[^ ^225^ ^]^ It was estimated that the wear debris related damage accounted for 4–5% of the implant failure 6–7 years after implantation.^[^ ^226^ ^]^

**Figure 14:**
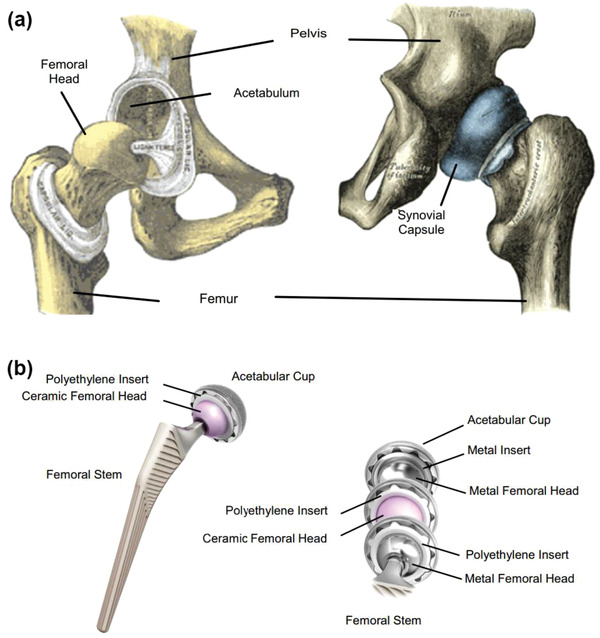
a) Anatomies of the hip joint. b) Examples of artificial joints illustrating total hip replacement components with corresponding materials. Left: ceramic‐on‐polyethylene structure; right: examples of metal‐on‐metal, ceramic‐on‐polyethylene, and metal‐on‐polyethylene systems. Reproduced with permission.^[^ ^141^ ^]^ Copyright 1995, Elsevier. Reproduced with permission.^[^ ^227^ ^]^ Copyright 2010, Elsevier.

Cobaltchromium‐molybdenum and ceramics combined with hard coatings are commonly used in artificial joints, for example, in the hip replacement shown in **Figure** **14**,^[^ ^14^, ^227^ ^]^ owing to their high wear and corrosion resistance as well as excellent strength and fracture toughness. Additionally, Ti alloys are applied in joint replacement due to their high corrosion resistance, good biocompatibility, desirable elastic modulus, and density similar to that of human bones.^[^ ^228^ ^]^ High friction and wear were observed in Ti alloys when they were used as articulating surfaces combined with ultrahigh‐molecular‐weight‐polyethylene (UHMWP) acetabula.^[^ ^228^ ^]^ A possible solution is to optimize the structural design of the joint systems that composed of Ti alloys at the bone interface and CoCr alloys on the articulating surfaces, namely metal‐on‐metal bearing, for example, the femoral head in Figure 14b.^[^ ^14^, ^227^ ^]^ A part of the bearing system was reported to show unexpected failure rates, particularly in women.^[^ ^14l^ ^]^ Mechanisms of this clinical observation of high failure rates are still unknown and thus remain open to researchers.

Being the most important properties in biotribological applications, the outstanding wear resistance and desirable wettability of ceramics make their use attractive in artificial joints. CoC, which was reported to have a hundredfold reduction of fracture^[^ ^229^ ^]^ and a survival rate up to ≈94% in femoral components after 17 years in vivo,^[^ ^230^ ^]^ demonstrated to be a promising ceramic material for joint replacement.

To further improve the wear resistance of artificial joints with prolonged lifetime and good biocompatibility, many novel coatings have been considered, such as DLC,^[^ ^231^ ^]^ ZrN,^[^ ^232^ ^]^ ZrO,^[^ ^233^ ^]^ TiN,^[^ ^234^ ^]^ TiC,^[^ ^235^ ^]^ and NbN^[^ ^236^ ^]^ coatings. More comprehensive reviews of recent progress on the applications of wear‐resistant materials in joint replacement may be referred to refs. ^[^ ^14l^ ^]^ and ^[^ ^227^ ^]^, where experiments and simulations were systematically discussed.

Our teeth composed of enamel, dentin, and cementum show high mechanical strength and hardness owing to their hierarchical‐structured mineralized tissues, which consist of nanocrystalline carbonated hydroxyapatite and water molecules.^[^ ^237^ ^]^ The percentage volume mineralized is 45–50% in dentin^[^ ^238^ ^]^ and ≈95% in enamel.^[^ ^239^ ^]^ As wear causes tooth degradations, which permanently exist during masticating, dental restorative materials with high wear resistance should be applied. Among all the restorative materials, composites and ceramics that have demonstrated outstanding wear resistance and hardness as well as the potential in aesthetic design are of the utmost interest.

The application of resin‐based composites in dental practices is considerably motivated by their good bonding to teeth and composition free of mercury.^[^ ^240^ ^]^ The composites consist of the dimethacrylate matrix and reinforcements of glass, quartz, colloidal SiO_2_, and ZrO_2_. The matrix property, additive content and property as well as the interface bonding between additives and the matrix strongly influence wear behaviors of the composites.^[^ ^241^ ^]^ The anti‐wear behavior of glass‐reinforced resin composites was reported by Arsecularatne et al.^[^ ^242^ ^]^ after reciprocating in vitro sliding wear tests. Fatigue wear was observed in the resin composites reinforced by alumina silicate glass or strontium glass characterized by second‐phase particle debonding and crack propagation along the interface beneath the surface layer, whereas abrasive wear was found in those reinforced by silica showing the pulling‐out of the second‐phase particles. Recent studies demonstrated that nanocomposites exhibit better wear resistance and chemical stability. However, mechanisms responsible for the better performance remain unknown.

The desirable anti‐wear performance, high chemical stability, good biocompatibility, and great potential in the aesthetic design of ceramics make them good candidates of surface coatings or the all‐ceramic restoration bulk for dental materials.^[^ ^243^ ^]^ Microstructures, fracture toughness, surface integrity, contact stresses, and working environment are the dominating factors determining their wear resistance. Previous research works demonstrated no significant wear dependence of dental enamels on hardness.^[^ ^244^ ^]^ Their wear resistance was more dependent on the ceramic microstructure and roughness. Generally, the reduced wear of the ceramics can be obtained by the increase of flexural strength and fracture toughness,^[^ ^245^ ^]^ addition of homogeneously‐distributed nano‐particles,^[^ ^246^ ^]^ reduction of microstructural defects (e.g., micro‐cracks and micro/nano‐voids),^[^ ^247^ ^]^ and by the polished surface.^[^ ^248^ ^]^

**Figure 15:**
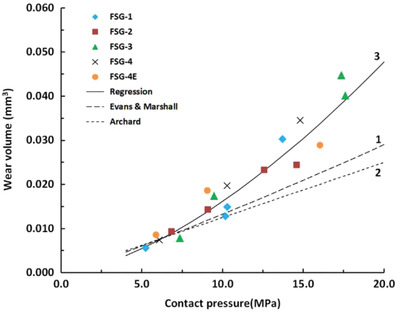
Experimental and numerical simulation results of the wear volume, where dotted lines 1 and 2 represent the expected wear volume obtained based on the numerical model and the Archard relation, respectively; solid line 3 denotes the fitted curve for the experimental wear volume. Reproduced with permission.^[^ ^249^ ^]^ Copyright 2015, Elsevier.

Thus far, most of the reported studies regarding applications of wear‐resistant ceramics in teeth have been focusing on experimental observations; only a few efforts in the modeling of ceramic wear were carried out. Arsecularatne et al.^[^ ^242^, ^249^ ^]^ proposed a model to describe the relationship between the wear volume *V* of dental ceramics and applied pressure *P* as*V* = 9.83 × 10^−4^ *P* ^9/8^. Based on the existing experimental observations, specific results were calculated by the above model and by the Archard relation as shown in **Figure** **15**.^[^ ^249^ ^]^ The underestimated wear of ceramics can be found in the results obtained by these models, typically at higher contact loads, where the local microstructural change affected wear but was not counted within the models. Further research efforts in the development of numerical models for theoretical descriptions of wear behaviors with underlying mechanisms are required for dental ceramics.

## 5 Conclusion and Outlooks

### 5.1 Concluding Remarks

A review of achievements in the wear reduction from aspects of novel design strategies for the surface and matrix, intrinsic material properties, and wide applications has been provided. Surface engineering including the design of coatings, surface texturing, surface hardening, and architecture effectively improves the wear resistance of surfaces by the introduction of a surface layer with higher hardness than the substrate or by the formation of a lubricating structure. Nanocomposite coatings, amorphous coatings, 2D material coatings, amorphous/nanocrystalline coatings, and gradient multilayer coatings exhibit exceptional wear resistance at specific working conditions such as corrosion, water lubrication, and electrical contact. The proper design of surface coatings with certain textures helps to achieve outstanding anti‐wear properties of surfaces at high contact stresses owing to the integrated effect of the wear‐resistant coating and lubricous texture. The surfaces with hierarchical structures substantially decrease wear, which was three orders of magnitude lower than that of the unprocessed ones.^[^ ^123^ ^]^ The strong in‐plane covalent bonds, ultrathin thickness, as well as high mechanical strength and flexibility of 2D nanomaterials make them the promising components in hierarchical anti‐wear structures.

Several strategies on the wear resistance enhancement of bulk materials without sacrificing their mechanical or electrical properties have been confirmed to be feasible, which mostly involved the change of microstructures and phase compositions of the bulk. For example, the transformation from fcc to hcp crystal structures by tuning the Al content resulted in a substantial increase in the hardness of the AgAl alloys owing to the decreased slip systems of hcp phases.^[^ ^135^ ^]^ Specific attention would be paid to the anti‐wear effect of carbon nanomaterial reinforcements that is highly dependent on their dimension, morphology, and content. MD simulations revealed that a strengthening effect was caused by the addition of graphene in polymers because the van der Waals interactions between the graphene and polymer matrices restricted the surrounding polymer chains.^[^ ^147^ ^]^ More detailed information on the effect of carbon nanomaterial reinforcements on different types of bulk materials including ceramics, metals, and polymers can be referred to our previous review studies.^[^ ^59^, ^60^ ^]^

Hardness is widely accepted as a significant parameter in determining the wear resistance. Very limited data are available on the assessment of the wear resistance using other critical intrinsic material properties including stiffness, strength, and cyclic plasticity. The wear resistance of the bulk can be increased by controlling its hardness, fracture behavior, plasticity, and strength that are influenced by the lattice distortion, bonding strength, grain sizes, precipitation, grain boundaries, dislocations, or twins. The wear resistance of metallic materials with an average grain size above 10 nm was in consistency with the classical Archard theory.^[^ ^138^ ^]^ However, a prominent deviation from the Archard theory was observed at the average grain size below 10 nm. This deviation was attributed to the local hardening of the worn surface caused by the grain growth and grain boundary relaxation during the repetitive sliding. Substrate stiffness plays different roles in the surface wear resistance in macro‐ and nanoscale domains. A preliminary relation between the polymer wear and ratio of the maximum contact stress to yield strength was obtained, which would be applied in the assessment of polymer wear resistance. The wear resistance of ceramic–ceramic, ceramic–metallic, and metallic–metallic tribological systems presented dependence on the *H*/*E* ratio, which has been confirmed in Al_2_O_3_‐CrN, Al_2_O_3_‐TiC, and steel‐steel sliding systems.^[^ ^179^ ^]^

### 5.2 Current Challenges and Future Perspectives

Remarkable advances of numerous innovative wear‐resistant materials in fundamentals and applications bring prospective challenges that will be of particular interest in the future research on energy savings. The large‐scale elaboration of nanostructures or phase compositions through the parameter optimization of preparation and post‐treatment processes would be an effective strategy to further enhance the material wear resistance, which focuses on the formation of nano‐twins, heterogeneous or gradient nanostructures, homogeneously‐distributed in situ precipitation, cryogenic deformation, and uniform dispersion of 2D nanomaterials in matrices with good interfacial compatibility. Computational modeling, typically the ab initio calculations and MD simulations, provides theoretical descriptions together with underlying mechanisms of nanostructural features for structural design and phase transformation. The reproducibility and stability of the designed nanostructures or phase compositions are the critical factors in the design of large‐scaled wear‐resistant nanostructures or phase compositions.

Over the past decade, research attention in AM has increased exponentially on the fabrication of lightweight and high‐performance structures from polymers, ceramics and metals. Limited data are directed toward the wear behavior analysis of the AM materials. The anti‐wear assessment of AM materials thus requires tremendous research efforts. Of particular interest are the AM metallic materials with nanocrystalline and nano‐twinned structures, AM amorphous materials, and AM high‐entropy alloys. Their outstanding mechanical properties have been demonstrated and shown great potential in anti‐wear applications.

Superlubricity has become increasingly important to minimize the negative effects of friction and wear, whereas the fabrication of superlubric surfaces has only been stabilized at the micro‐ and nanoscale under specific surface conditions or at the macroscale for a period of several minutes. Recently, 2D materials show great potential in extending superlubricity to reliable practical applications, depending on their structures and sizes. The efficient use of 2D material‐wrapped nanoparticles would allow superlubricious contact to be maintained at extended timescale conditions. Further experimental identifications, modeling simulations, and theoretical descriptions of 2D materials are required. They focus on the fabrication of materials with large sizes but low‐defect density, development of heterostructures, and modification of materials with functionalized groups.

Materials applied at elevated temperatures suffer from severe wear damage. The microstructures can hardly be experimentally characterized in situ during the sliding process. MD simulations deal with micron‐sized systems that are large enough to cover grains, where one or a combination of deformation mechanisms including the dislocation slip, grain boundary migration, and twinning take place. Their atomistic‐level resolution allows understandings into the interplay between dislocations and grain boundaries responsible for wear resistance. The simulations would then be promising to reveal relations between the change in microstructures at high temperatures and the wear behavior, which are critical for high‐temperature wear‐resistant materials.

It is hoped that the numerous achievements and the prospective challenges would boost more research interest in this field and make a significant impact on fundamental material science to provide highly wear‐resistant materials in a broad range of advanced applications.

## Conflict of Interest

The authors declare no conflict of interest.
